# Biofilms in Surgical Site Infections: Recent Advances and Novel Prevention and Eradication Strategies

**DOI:** 10.3390/antibiotics11010069

**Published:** 2022-01-07

**Authors:** Andriy Hrynyshyn, Manuel Simões, Anabela Borges

**Affiliations:** LEPABE—Laboratory for Process Engineering, Environment, Biotechnology and Energy, Faculty of Engineering, University of Porto, Rua Dr. Roberto Frias, s/n, 4200-465 Porto, Portugal; up201909519@edu.fe.up.pt (A.H.); mvs@fe.up.pt (M.S.)

**Keywords:** biofilms, surgical site infections, multidrug-resistant bacteria, nanoparticles, phytochemicals

## Abstract

Surgical site infections (SSIs) are common postoperative occurrences due to contamination of the surgical wound or implanted medical devices with community or hospital-acquired microorganisms, as well as other endogenous opportunistic microbes. Despite numerous rules and guidelines applied to prevent these infections, SSI rates are considerably high, constituting a threat to the healthcare system in terms of morbidity, prolonged hospitalization, and death. Approximately 80% of human SSIs, including chronic wound infections, are related to biofilm-forming bacteria. Biofilm-associated SSIs are extremely difficult to treat with conventional antibiotics due to several tolerance mechanisms provided by the multidrug-resistant bacteria, usually arranged as polymicrobial communities. In this review, novel strategies to control, i.e., prevent and eradicate, biofilms in SSIs are presented and discussed, focusing mainly on two attractive approaches: the use of nanotechnology-based composites and natural plant-based products. An overview of new therapeutic agents and strategic approaches to control epidemic multidrug-resistant pathogenic microorganisms, particularly when biofilms are present, is provided alongside other combinatorial approaches as attempts to obtain synergistic effects with conventional antibiotics and restore their efficacy to treat biofilm-mediated SSIs. Some detection and real-time monitoring systems to improve biofilm control strategies and diagnosis of human infections are also discussed.

## 1. Introduction

Surgery is the main cause of most hospital-acquired infections, injuries, accidents, invalidity, and death in the global healthcare system. Postoperative wound infection is a common healthcare problem among surgically treated patients. The development of a surgical site infection (SSI) is due to microbial contamination of the surgical wound, which may come from either endogenous or, less frequently, exogenous sources [1]. SSIs can sometimes be superficial infections involving the skin or lead to more serious outcomes, affecting the tissues under the skin, organs, or the implanted material. In general, when the microbial concentration is higher than 10^4^ microorganisms per gram of tissue there is a potentially high risk of an infected wound [2].

According to the Centers for Disease Control and Prevention (CDC) in the United States (US), the rate of surgical procedures is considerably high every year [3]. In 2011, a total of 11.2 million inpatient surgical procedures were performed in the US hospitals, whereas, in 2014, these procedures constituted a total of 14.2 million surgeries performed in the inpatient settings [4,5]. Moreover, a CDC healthcare-associated infection prevalence survey found that around 110,800 SSIs related to inpatient surgical procedures occurred in 2015 [3]. Furthermore, in line with the annual epidemiological report for 2017, retrieved from the European Centre for Disease Prevention and Control (ECDC) data storage, 648,512 surgical procedures were performed in 1,639 hospitals across the European Union (EU) and the European Economic Area (EEA). During this period, 10,149 SSIs were reported, with the percentage of SSIs varying from 0.5% to 10.1%, depending on the type of surgical procedure [6].

Despite the advances made in infection control practices, including the improved operating room ventilation, sterilization methods, barriers, surgical techniques, and availability of antimicrobial prophylaxis, SSIs remain a significant cause of morbidity, prolonged hospitalization, and death. More specifically, SSIs are responsible for a mortality rate of 3%, and 75% of SSI-associated deaths are directly attributable to SSI. Therefore, SSIs prevail as one of the most substantial economic burdens on the healthcare system, with an estimated annual cost of 3.3 billion US dollars [6]. It must be noted that the National Institutes of Health (NIH) has proposed that approximately 80% of SSIs reported in the US may be related to the formation of microbial sessile communities, known as biofilms [7].

This review aims to provide an overview of the biofilm-related SSIs, as well as the most recent healthcare achievements to preclude or treat these infections. Special focus will be given to strategies involving the use of nanoparticles (NPs) and molecules from plant metabolism (phytochemicals). Moreover, diagnosis and monitoring systems for detection of biofilm infections are described, including the use of artificial intelligence and ultrasound-assisted strategies, as well as biofilm control advances made with synthetic biology approaches.

## 2. Biofilms in SSIs

**Figure 1:**
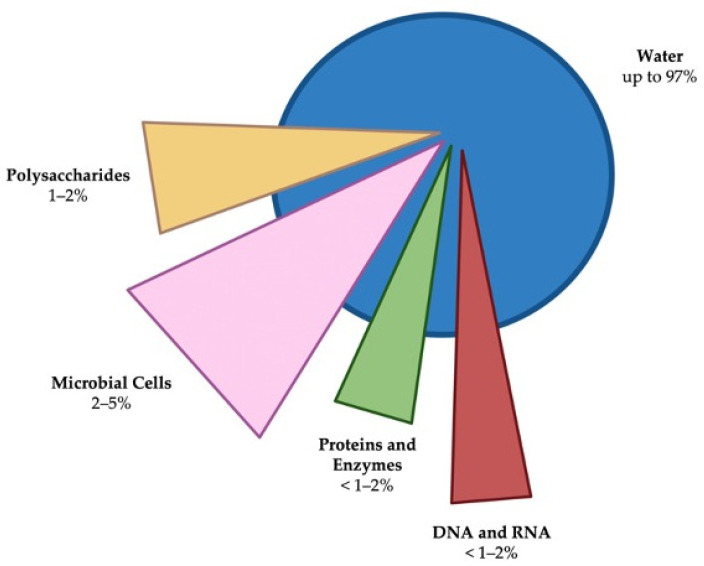
Major components of a biofilm and their typical levels.

Biofilms are defined as complex three-dimensional communities of microorganisms usually found attached to inert or living surfaces and encased within a self-produced protective matrix of extracellular polymeric substances (EPS). The biofilm is essentially composed of water, microbial cells, and EPS, including polysaccharides, proteins, lipids, extracellular enzymes, metal ions, and nucleic acids such as extracellular DNA [8,9]. Figure 1 illustrates the main constituents of a biofilm.

These constituents, in addition to securing the biofilm to the surface, allow the capture of nutrients, provide structural support, and protect the biofilm from external stresses. Typically, biofilms can remain unperturbed by antimicrobial or neutrophil attacks and can survive in relatively harsh environments. Thus, the almost invulnerable nature of biofilms delays healing without inducing a dramatic host response [10]. In addition to the aforementioned aspects, the EPS is the key to maintaining the proximity of the cells in the communal ecosystem, thereby enabling cell-to-cell communication, also known as quorum sensing (QS), and serves as an adjuvant in the exchange of genetic material through horizontal gene transfer [8].

### 2.1. Biofilm-Forming Bacteria Associated with SSIs

Biofilms may comprise bacteria of the same or from different species. Depending on the procedure performed, some of the most common endogenous microorganisms associated with SSIs are *Staphylococcus aureus*, coagulase-negative staphylococci, *Enterococcus*, and *Escherichia coli*. For instance, in cardiac, ophthalmic, orthopedic, breast, and vascular surgeries, the most common causative organisms are *S. aureus* and coagulase-negative staphylococci, while in abdominal surgeries, Gram-negative bacilli and anaerobes are more common. On the other hand, exogenous sources of microorganisms are usually found in the operating room environment, including air, surgical instruments, materials, and staff members. The most common exogenous microorganisms are staphylococci and streptococci [11]. For example, surgical personnel colonized with *S. aureus* or carriage of group A streptococci (*Streptococcus pyogenes*) by operating room personnel have been implicated as causes of several SSI outbreaks [12,13].

The microorganisms residing within the microbial biofilm community are both phenotypically and genetically different from their free-living “planktonic” counterparts. More precisely, planktonic microorganisms are those that have been commonly studied during standard laboratory research and antibiotic sensitivity testing. In contrast, bacteria in the sessile state have distinctive physiological and biochemical properties as opposed to planktonic bacteria. As an example, bacteria residing within biofilms are known to be regulated by diffusible molecules, or pheromones, which aid in the expression of proteins of individual bacterium, providing them with enhanced survival strategies [10].

### 2.2. Biofilm Recalcitrance to Antimicrobial Treatments

The majority of antimicrobial treatments currently available were generally developed and tested on planktonic bacteria. As a result, these treatments are frequently ineffective against pathogenic biofilms, which can be up to 1,000 times more tolerant to antimicrobial treatments. This phenomenon of biofilm recalcitrance makes them incredibly difficult to treat and eradicate successfully [8]. The biofilm acts as a physical barrier that reduces the rate of penetration of antibiotics, antibodies, and granulocytic cell populations. Antimicrobial tolerance is mediated by several mechanisms, most of which are related to phenotypic alterations and multi-cellularity, rather than the type of genetic adaptation responsible for antibiotic resistance of the cells under planktonic conditions. Besides the transfer of resistance genes between neighboring bacteria and QS, as previously mentioned, the growth rate is a significant determinant of bacterial susceptibility to many antimicrobial agents, even in planktonic cells [14]. The EPS matrix is critical since immobilization can cause phenotypic heterogeneity of the cellular growth rate within the biofilm due to localized depletion of nutrients and oxygen. For this reason, some bacteria that are not dividing develop “drug-indifference” to certain antimicrobial agents. Another significant aspect affecting biofilm recalcitrance is the presence of “persister cells”, which are phenotypic variants that did not result from stable genetic alteration and are essentially indifferent to antimicrobial treatments with later proliferation [14]. Thus, acknowledging the presence of biofilms as a potential cause of SSIs may explain unsatisfying responses obtained from traditional approaches, such as promoting drainage, systemic drug-therapy (e.g., antibiotics), or delayed closure [10]. New strategies for the prevention, removal, and complete eradication of microbial biofilms are urgently required to preclude or treat biofilm-associated infections [8].

## 3. Prevention of SSIs

Since SSIs lead to adverse patient outcomes, including prolonged hospitalization and death, several rules and guidelines must be applied to prevent them. It has been estimated that each patient with an SSI requires at least additional six days of hospitalization, thus doubling hospital care costs [15]. However, approximately 40–60% of SSIs are preventable with the appropriate use of prophylactic antimicrobial agents [16].

**Figure 2:**
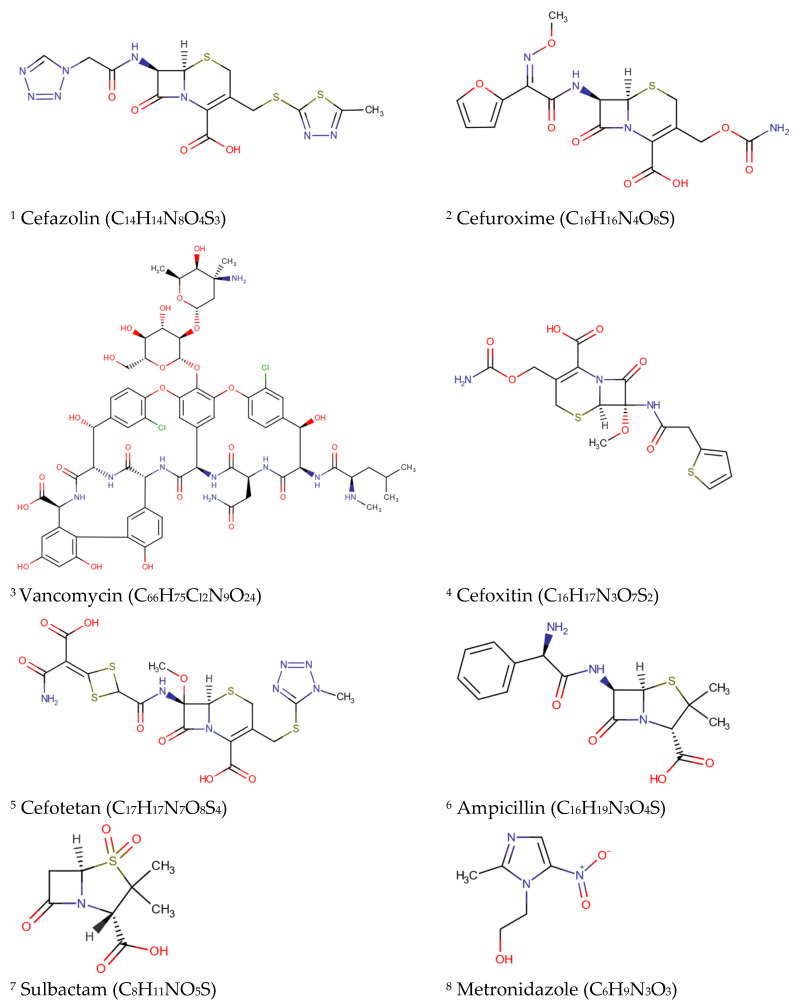
Chemical formulas/structures of recommended antibiotics for prophylactic prevention of SSIs (obtained from DrugBank; https://go.drugbank.com/drugs) (accessed on 9 September 2021).

According to the guideline for the prevention of SSIs proposed in 2017 by the Healthcare Infection Control Practices Advisory Committee (HICPAC), a federal advisory committee to the CDC, several measures must be performed to guarantee significant reduction of wound infections. These include the following: (1) the patient must be well-prepared and informed about the operation and infection prevention measures; (2) it is imperative to ensure that the patient does not have signs of ongoing infections, and if the patient does they need to undergo eradication of the infection before admission; (3) preoperative surgical site skin disinfection and hair removal should be appropriate for the location and type of procedure (clipping is preferred, as shaving causes skin damage and increases the risk of infection); (4) operating room sterility rules must be followed; and (5) peri- and postoperative administration of prophylactic antibiotics and appropriate wound dressings must be guaranteed for the specific procedure [17]. Moreover, for patients undergoing cardiac surgery, postoperative morning blood glucose levels should be controlled (200 mg per dL [11.10 mmol per L] or less), and in cases of colorectal surgery, patients should be normothermic (36 °C [96.8 °F] or greater) within the first 15 min after leaving the operating room [18]. Other aspects, such as improving the patients’ natural defense mechanisms by early mobilization and improving their nutritional status, are important factors affecting the pace of recovery. Table 1 shows recommended antimicrobials (see Figure 2 for chemical structure) for prophylactic regimens administered to prevent SSIs in different types of surgical procedures. Essentially, basic support by hospital leaders, knowledge and skills of the surgical teams, availability of resources, excellent treatment of the complete patient admission, and monitoring patients after discharge may lead to the prevention of SSIs, lower death rates, and less expense for the healthcare system [1].

## 4. Conventional Treatment and Management of Biofilm-Associated SSIs

SSIs can be divided into three main types: superficial incisional, deep incisional, and organ/space or intracavitary. Superficial SSIs are easier to treat and usually require only simple opening and drainage, whereas deep incisional SSIs typically require more thorough surgical debridement and often adjuvant antibiotic treatment. Intracavitary SSIs also often require surgical intervention [19].

The above-mentioned SSIs are tissue-based infections, which differ from device-related infections caused by the microbial colonization of implanted medical devices. In tissue-based infections, surgical debridement is usually performed, which consists of the removal of necrotic (devitalized) or infected skin tissue to promote wound healing. This procedure is essential for chronic wound infections, as those wounds are trapped in the first stage of healing and show no significant progress towards the resolution of the infection. Frequent debridement of the surface of the wound forces constant reconstitution of the biofilm, making it more susceptible to topical and systemic antibiotics and appropriate biocides. In addition, negative pressure wound therapy (NPWT) is also recommended for patients with an SSI. A special dressing or bandage is sealed over the infected site and a gentle vacuum pump is attached, which draws out fluid and infection from the wound and helps it to heal by promoting the growth of new tissue. These procedures are followed by irrigation, preferably with an antiseptic agent, and then parenteral antibiotics are administered [14].

On the other hand, device-related infections are caused by the colonization of microorganisms during the implantation processes, constituting a risk to the patient’s wellbeing and compromising the device function. Examples of these devices include central and peripheral vascular catheters; tissue fillers and breast implants; endotracheal tubes; contact lenses; orthopedic and prosthetic implants; urinary catheters; and cardiac implants such as pacemakers, vascular grafts, or cardiac valves. In cases of delayed or late infections, the implanted device or material is usually removed to ensure that the biofilm is eradicated, followed by the insertion of antimicrobial adjuncts, such as antimicrobial spacers, beads, or sutures, together with parenteral antibiotics. This two-stage surgical procedure has a success rate of 93–100% and comprises the removal of the infected device with debridement of the devitalized tissue and placement of an antibiotic-impregnated filler in the wound. Regarding the antibiotic therapy for the treatment of these biofilm-mediated infections, it is frequently a combination therapy of rifampin, a fluoroquinolone, followed by a glycopeptide. Alternative options in the combination therapy include linezolid, daptomycin, tigecycline, cephalosporins, carbapenems, amoxicillin, and sulfamethoxazole-trimethoprim [20].

**Figure 3:**
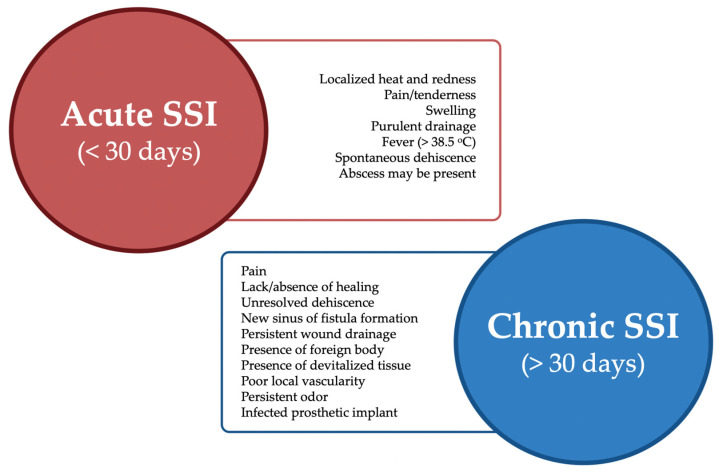
Major differences between acute and chronic SSIs.

It is also important to note that SSIs can be described as acute (<30 days) or chronic (>30 days) wound infections (Figure 3). Acute wound infections, caused by free-floating bacteria, tend to be progressive with rapid manifestation and tissue destruction, but usually heal within a predictable and expected rate of a normal wound healing process. Chronic infections follow a persistent undulating course with frequent exacerbations and will generally respond incompletely to systemic antibiotics, often reemerging once the treatment plan is withdrawn. Therefore, as a sole strategy, topical and systemic antibiotics are unable to successfully manage biofilm phenotype bacteria and should be combined with other approaches [10]. Moreover, chronic infections are frequently associated with monomicrobial biofilms at early stages, which further develop into more complex polymicrobial infections. If this occurs, polymicrobial infections decrease wound healing more significantly than monomicrobial biofilms, mainly due to synergistic interactions among bacteria. This can be attributed to different mechanisms, particularly the expression of virulence factors [21].

## 5. Novel Strategies to Control Biofilm-Associated SSIs

Currently, the survival of biofilm-forming bacteria and the emergence of new resistant bacterial infections (i.e., infections caused by multidrug-resistant and biofilm-producing *Acinetobacter baumannii*, *E. coli*, *Klebsiella pneumoniae*, *Pseudomonas aeruginosa*, *S. aureus*, and other microorganisms) pose a serious threat to public health and have created the need for novel antimicrobial and antibiofilm treatment strategies. Some of these strategies that are being presently adopted to treat SSIs associated with biofilm formation are: inhibiting the attachment of the microorganisms to the substratum, using special compounds that interfere with and unsettle the biofilm structure, and disrupting the biofilm at the initial stages [22,23]. For example, to help control the rate of SSIs, new antiadhesive surfaces with altered physical, chemical, and topographical properties that prevent microbial adhesion and thereby biofilm formation have been tested on several medical devices [24,25,26,27,28,29,30,31,32,33,34].

**Figure 4:**
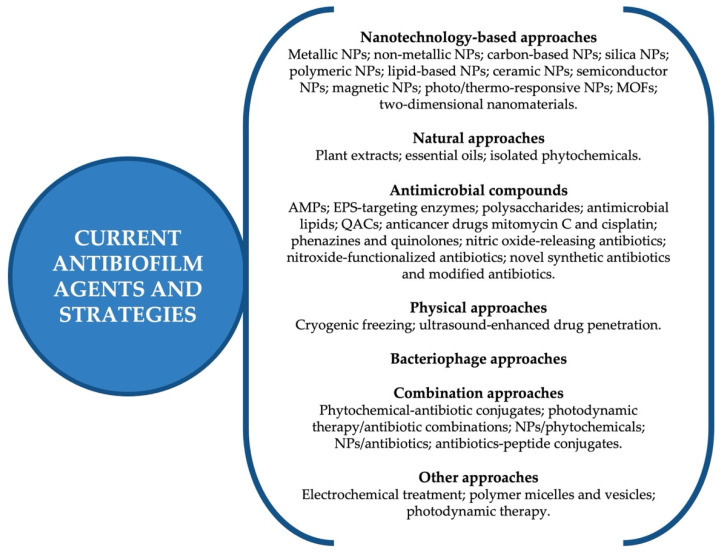
Antibiofilm agents and strategic approaches that are currently being explored and implemented in attempts to fight biofilms in SSIs.

Other approaches along with surface modifications have also been explored and focused mainly on compounds that interfere with QS by delivering signal blockers, hindering the production of functional bacterial adhesins, inducing biofilm detachment, and interfering with biofilm regulation mechanisms [28,35,36,37,38,39,40,41,42]. Figure 4 details the antimicrobial and more importantly antibiofilm agents and strategic approaches that are currently being explored in several studies to help control biofilm-associated SSIs. Several antimicrobial compounds have been identified as potential biofilm eradicators, namely antimicrobial peptides (AMPs), EPS-targeting enzymes, antimicrobial lipids, quaternary ammonium compounds (QACs), nitric-oxide-releasing antibiotics, and others [8]. Nanotechnology-based approaches, predominantly different types of NPs, metal organic frameworks (MOFs), and other nanomaterials, are also amongst the most studied line of attack to deal with pathogenic biofilms [22,24,25,26,27,28,29,30,31,32,35,36,43,44,45,46,47,48,49,50,51,52,53]. Natural plant-based products are being developed to help overcome the problem with multidrug-resistant bacteria, namely plant extracts and isolated compounds, as well as essential oils that contain large amounts of phytochemicals [33,34,37,38,39,40,41,42,54,55,56,57,58,59,60,61,62,63,64,65]. In addition, physical approaches (cryogenic freezing, ultrasound), bacteriophages, electrochemical treatments, photodynamic therapy (PDT), combination approaches, and other strategies are also among a few other examples that are being presently implemented [26,30,35,36,44,52,54,60,61,62,63,64,65,66,67]. For example, the use of antimicrobial PDT, has been extensively studied over the last decade. PTD combines non-toxic dyes called photosensitizers (e.g., porphyrins, ruthenium complexes) with harmless visible light, thereby forming highly toxic reactive oxygen species (ROS) that exhibit considerable antimicrobial activity against a vast range of microbes, suggesting a promising alternative to conventional antibiotherapy [68,69,70,71,72,73].

Only a few in vivo (preclinical) or clinical trials have demonstrated better treatment of biofilm infections, despite several in vitro research studies demonstrating successful results in terms of antibiofilm treatment. In vitro models are essential for understanding the molecular mechanisms of biofilm establishment and development as well as their role in the infectious process. However, the results of in vitro studies of biofilm development in clinical isolates have not always matched the findings of in vivo investigations. This might be due to the limited relationship between in vitro and in vivo biofilm formation, unclear function and influence of the biofilm in the infection process, or lack of understanding of biofilms’ role in health and disease contexts [23]. That being said, cost efficient alternatives are still lacking and more studies are required in the field of in vivo molecular mechanisms.

This review will discuss the most recent studies that have been conducted for two of the main antibiofilm approaches: the use of NPs and phytochemicals (see Supplementary Materials Table S1). These strategies are increasingly attracting the attention of many researchers, allowing evolution of the knowledge necessary to overcome the issue of SSIs caused by highly resistant biofilm-forming bacteria, mainly in two possible ways: contamination of either the surgical site or the implanted devices during surgical procedures.

### 5.1. Nanotechnology-Based Strategies

Nanotechnology-based approaches, specifically functionalized NPs, have recently been investigated to be used against bacterial biofilm-mediated SSIs due to their strong bactericidal and antibiofilm properties.

It is known that sutures are the most used surgical implants, accounting for 57% of the overall surgical equipment market. It has also been confirmed that surgical sutures are particularly vulnerable to microbial colonization and biofilm formation, and hence have the potential to cause microbial infections in addition to common foreign body reactions. To minimize the incidence of wound infections, particularly SSIs, recent studies emphasized the design of antibiotic-coated sutures. In vitro laboratory testing, in vivo animal trials, and clinical studies have all demonstrated that these sutures have effective antibacterial action [24]. More precisely, sutures enhanced with levofloxacin and ciprofloxacin showed inhibitory and bactericidal efficacy against *E. coli*, whereas chlorhexidine and octenidine significantly reduced *S. aureus* adherence [25]. However, the rising incidence of antibiotic resistance, as well as the cytotoxicity of these compounds in higher doses, demands alternative and effective control strategies. For this reason, NPs are currently deemed to be an appealing approach for biofilm control due to their capability to destroy planktonic bacteria, prevent biofilm formation, and penetrate and disintegrate already formed biofilms [74].

Recent advances in nanotechnology have allowed researchers to conclude that these particles have unique mechanisms of antibacterial activity as compared to standard antimicrobial agents and have given assurance that they can prevent antibiotic-resistant biofilm infections. Because of their characteristics such as a small size, shape, surface charge, and composition, NPs may easily penetrate microbial cell walls and biofilm layers, causing permanent damage to cell membranes and DNA, as well as oxidative damage and formation of free radicals. Furthermore, properties like a long plasma half-life and high surface-to-volume ratio make them potential candidates for effective drug loading and targeting entities [22].

Recent studies have reported the broad-spectrum antibacterial effects of silver NPs (AgNPs), which are a particular type of metallic NPs [75,76,77,78]. When compared to antibiotics that are currently being used against pathogenic bacteria, silver works by binding to the cell membrane, changing its structure, and triggering rupture and lysis. Thus, products designed by nanotechnology and coated with AgNPs are the fastest-growing segment for most industries and several medical applications are arising from their antimicrobial features. Examples of such advanced products already available on the market include biomedical devices, surgical instruments, contraceptive devices, wound dressings, and bone prostheses [24]. For example, in Baygar et al. [24], non-absorbable silk sutures were coated with biologically synthesized AgNPs that were obtained via an ecologically conscious, non-toxic, and cost-efficient method using *Streptomyces griseorubens* cultures. AgNPs strongly adhered to the sutures and exhibited significant antimicrobial capacity against pathogenic microorganisms, such as *Candida albicans*, *E. coli*, and *S. aureus*. Despite the increasing Ag^+^ ion release from the degradation process of the sutures, the silver levels assessed were below the toxicity limits, suggesting that it would not affect the patient’s cell viability in clinical applications or the wound healing process [24].

It must also be noted that biosynthesized AgNPs have an advantage over conventionally synthesized AgNPs because the compounds involved in the synthesis may enhance the antimicrobial activity of the NPs [79]. Syukri et al. [25] demonstrated that coating silk sutures with AgNPs, which used *Eucalyptus camaldulensis* as a capping and reducing agent for the biosynthesis of AgNPs, improved the tensile strength and reduced the average roughness when compared to uncoated sutures. In addition, these coated sutures were biocompatible with adult human keratinocyte cells and exhibited strong bacteriostatic and bactericidal activities against tested wound pathogenic bacteria. These effects occurred mainly through attachment of AgNPs to cell membrane and penetration into the cytoplasm, with consequent cell lysis. The authors concluded that antibacterial AgNP-coating of surgical sutures may be employed to successfully prevent SSIs caused by bacterial biofilm formation [25]. Edis et al. [26] investigated combinations of phytochemicals including *trans*-cinnamic acid, *Cinnamomum zeylanicum* bark extract, and povidone-iodine to be used as reducing and capping agents to biosynthesize AgNPs and increase their antimicrobial effect. While *trans*-cinnamic acid causes damage to the microbial cell membranes on its own, it also enhances the release of Ag^+^ ions, which cause even more damage. The other two, povidone-iodine and *Cinnamomum zeylanicum* bark extract, present strong antimicrobial activity due to formation of free molecular compounds with known antimicrobial effects, such as iodine and cinnamic acid, respectively. The study focused on assessing the efficacy of these NPs as natural drug carriers and coating agents on surgical sutures against 10 different reference microorganisms associated with biofilm formation in SSIs (see Supplementary Materials Table S1). Overall, the results obtained allowed the researchers to conclude that sutures coated with these AgNPs have the potential to prevent biofilm anchoring and further development, thereby preventing SSI occurrence [26]. Another work by Syukri et al. [27] demonstrated the possibility of using AgNPs on non-absorbable material, such as nylon sutures, without altering its physical and mechanical properties, while exhibiting excellent antibacterial activity [27]. Puca et al. [28] explored the use of a silver-nanotech patented product, TIAB, which consists of microcrystalline titanium dioxide (TiO_2_) NPs covalently linked with monovalent Ag^+^ ions. This product is commercially available under the trademark Peonil^®^ and used as an antibacterial and antibiofilm agent for the treatment of SSIs in the male urogenital tract. For the study, a mixture of TIAB alongside *Aloe vera* extract and hyaluronic acid was applied on three commercially available and commonly used braided surgical sutures and exposed to different microorganisms (*S. aureus*, *Enterococcus faecalis*, and *E. coli*). The results presented in this work confirmed that surgical sutures coated with Ag^+^–TiO_2_ NPs have the potential to interfere with the microbial QS system, thereby affecting biofilms’ adhesion and formation post-surgery, which decreases the chance of developing an SSI. Moreover, the authors suggested that applying Peonil^®^ to suture threads in the form of a cream is also a valid strategy that avoids the use of coated sutures, which are more expensive and can induce side effects to patients, namely topical toxicity or allergy [28].

On the other hand, Xiang et al. [29] emphasized the need to upgrade the already existing AgNP-based wound dressings since these tend to get easily masked by absorbed conditioning films composed of proteins and dead microorganisms. This frequently results in loss of antibacterial active contact surface as well as leading to a more inflammatory response. For this purpose, *zwitterionic* AgNPs were synthesized using poly(carboxybetaine-co-dopamine methacrylamide) copolymer (PCBDA) as a reducing and stabilizing agent, and were then immobilized on amino-modified cotton gauze (CG) dressings. The results regarding the in vivo wound healing assay confirmed that this PCBDA@AgNPs-CG dressing not only effectively inhibited biofilm formation of *E. coli*, the *S. aureus* reference strain, and methicillin-resistant *S. aureus* (MRSA) isolates but also reduced inflammation and promoted wound healing [29].

A different application of AgNPs described by Ständert et al. [30] consisted of embedding AgNPs in a purpose-created amphora-shaped porous structure on titanium implants. What is so special about this structuring is that it creates hydrophilic surface conditions as well as capillary forces that allow the pores to be loaded with additional antibiotics, such as gentamicin used herein, enhancing the antimicrobial effect of the AgNPs. In fact, the combination of AgNPs and gentamicin creates a synergistic effect, in which AgNPs lead to ROS formation, while gentamicin targets and attacks the 30S ribosomal subunit, with both being detrimental to bacterial cells. Moreover, these implants with or without gentamicin-loading revealed good cytocompatibility, with no negative effects on human osteoblast-like cells, making them perfect candidates for future applications in orthopedic surgeries [30]. Surmeneva et al. [31] also addressed the problem of implant-associated SSIs by incorporating silver and calcium phosphate (CaP) NPs on the surface of Ti6Al4V alloy scaffolds used as material for the manufacturing of orthopedic implants. This nanocoating resulted in changes to the hydrophobicity and surface roughness, which inhibited *S. aureus* adhesion within the substrate. Moreover, while AgNPs hindered bacterial growth, CaP deposits showed positive effects on osteogenic cell regulation and bone regeneration [31].

Besides nano-based coating materials applied to implant surfaces and surgical devices, which mainly constitute a prevention approach to biofilm establishment, other eradication approaches were also implemented in several studies, and a few are currently being developed (see Supplementary Materials Table S1). For instance, Permana et al. [46] showed the feasibility of encapsulating AgNPs synthesized using green tea extract into bacteria-responsive microparticles (MPs) prepared from poly(*Ɛ*-caprolactone) (PCL) and decorated with chitosan to improve the adhesion to bacterial biofilms. These MPs were then incorporated into a delivery system of dissolving microneedles (DMNs), which significantly enhanced the penetrability of AgNP-loaded MPs through the biofilm, thereby improving antibiofilm activity against *S. aureus* and *P. aeruginosa* strains at the exact area of infection [46]. Moreover, in a previous study of Permana et al. [47], DMN-mediated delivery of NPs was assessed using different materials. The DMNs were made of doxycycline and the NPs were made from bacterially sensitive polymers, such as PCL and poly(lactic-co-glycolic acid) (PLGA), coated with chitosan. This approach resulted in improved biofilm penetration and release of doxycycline into the infection site, which enhanced the overall antibacterial and antibiofilm activities [47]. Mir et al. [35] used PCL-NPs loaded with the phytochemical carvacrol (CAR PCL-NPs) and evaluated their antibiofilm activity at the target site through the use of an in vivo MN liquid injection system for direct delivery of NPs [35]. CAR’s hydrophobic nature interacts with the bacterial cell membrane, causing loss of integrity, whereas its hydrophilic side enhances its diffusion through the biofilm layers [35]. This novel approach of assisted delivery of NPs showed to be more effective for enhanced site-specific accumulation of CAR and a prolonged antibiofilm effect, rather than the use of topical hydrogels to treat infections, as suggested in their previous work [36].

Other types of NPs and nanocomposites with potential antibacterial properties have been synthesized, and their potential as NP-based treatments of biofilm infections caused by biofilm-forming bacteria have been assessed (see Supplementary Materials Table S1). Gao et al. [48] studied the exposure of some pathogenic bacterial strains, particularly *S. aureus* biofilms, to polydopamine photothermal NPs (PDA-NPs) without any surface-functionalization, followed by near-infrared (NIR) irradiation. Two different modes of clinical infection treatments were investigated using in vitro models, namely eradication as well as prevention of the development of an existing infectious biofilm. The results obtained were more successful for the prevention model, confirming that already established biofilms pose a barrier to heat dissipation and penetration of photothermal NPs, which require surface modification to enhance the antibiofilm activity [48]. As a way of providing better photothermal therapy treatment, capable of removing pre-established biofilms without inducing damage to normal patient’s tissue, Zhang et al. [49] constructed unique pyramid-shaped chiral glutamic acid (^D^/_L_-Glu) functionalized gold nanobipyramids (AuNBPs). This conjugation of ^D^/_L_-Glu improved the targeting and interaction of AuNBPs with *Staphylococcus epidermidis* and *E. coli* bacterial strains, while the small size and sharp tips of AuNBPs promoted penetration and disruption of bacterial cells and the biofilm. Both in vitro and in vivo antibacterial and antibiofilm activity evaluations achieved remarkable results. Moreover, it was demonstrated that this approach could be a potentially efficient way of treating biofilm-associated SSIs, while avoiding the minimal cytotoxicity of normal tissues [49]. In another study, Kirui et al. [50] evaluated the use of gold NPs (AuNPs) in a AuNP-targeted pulsed laser therapy. By using this method, the authors observed that the photothermal destruction of the EPS matrix and cellular components of the biofilm potentiated the activity of gentamicin and amikacin antibiotics against MRSA and multidrug-resistant *P. aeruginosa* established biofilms [50].

Other studies were performed using unconventional NP-based strategies. For example, Reifenrath et al. [51] studied the development of implant-directed magnetic drug carriers, using nanoporous silica NPs as a promising strategy in overcoming the problem of implant-associated infections. In this approach, the NPs contained a superparamagnetic iron oxide core, allowing for targeted accumulation of NPs at the infection site, whereas the nanoporous silica shell could be useful to carry large amounts of the drug [51]. Kurniawan et al. [32] presented a solution for a common issue that is usually encountered in many hospital facilities, contaminated bed sheets, as well as other medical devices that are listed as potential sources of infection. This research group had the idea of using a multiple-layer technique of coating NPs with zinc oxide (ZnO-NPs), resulting in increased hydrophobicity of the bed sheets and good antibacterial activity against both Gram-positive and Gram-negative bacteria. These ZnO-NPs have the particularity of promoting ROS generation and Zn^2+^ ions’ release, which reduce the activity of Zn^2+^-dependent enzymes and transcription factors and/or cause lysosomal destabilization in the bacterial cells, leading to their death. Moreover, ZnO-NPs promote the production of H_2_O_2_ under ultraviolet light irradiation, which is severely toxic to living cells [32]. Kapustová et al. [52] developed eco-friendly nanosystems of encapsulated *Thymus capitatus* and *Origanum vulgare* essential oils in biocompatible PCL nanocapsules. They demonstrated that nanoencapsulation of essential oils increases their antibacterial, antifungal, and antibiofilm activities, while also significantly reducing the cytotoxicity to human keratinocyte cell lines. Therefore, this approach presented by the authors could be further explored as a potential ecological alternative in the development of new antimicrobial strategies for the healthcare system [52]. Shang et al. [53] synthetized exceptional sandwich-structured PDA colloidal particles coated internally and externally with AgNPs, which significantly enhanced the antibacterial performance against *E. coli* and *S. aureus* due to a more intense and lasting release of Ag^+^ ions. This model allows external AgNPs to suffer a quick and strong release of Ag^+^ ions, while internal AgNPs provide slow yet continuous antibacterial activity. In vivo studies showed that these particles were able to successfully inhibit biofilm formation and treat bacterial infections caused by *S. aureus*, through interactions with sulfide-groups within enzymes and proteins, causing structural changes and functional damage to cell membranes [53].

It is also important to mention that nanotechnology-based approaches include a vast variety of NPs and nanocomposites, including polymer-based NPs, dendrimers, liposomes, and others. There are already manuscripts that have reviewed the use of liposomes as well as polymeric NPs with inherent antibiofilm activity [80,81,82]. Polymeric NPs are the most successful NPs used as antibacterial and antibiofilm strategies since these composites have shown great potential for targeted delivery of drugs in the biomedical industry [80,81]. They present attractive properties, such as biocompatibility, non-toxicity to biological systems, biodegradability, stability during storage, controlled release, target delivery, and harmfulness to pathogenic microbes, resulting in higher therapeutic efficacy [83]. For example, in a research by Nie et al. [84] (see Supplementary Materials Table S1), poly(acrylic acid) capped iron oxide NPs were fabricated and their antibacterial and antibiofilm activities were assessed via magnetic force against *E. coli* and *S. aureus* (planktonic) as well as MRSA (biofilm). Under alternating current applied field conditions, the NPs adhered to the bacterial cells, entered the matrix, and released free radicals, then carried out a peroxidase kind of activity to cleave and damage the biofilm [84]. Another study by Porter et al. [85] focused on the development of self-assembled peptide nanostructures composed of a diphenylalanine (FF) motif. Although nanotubes are not necessarily defined as polymers, these structures exhibit, in fact, polymeric behavior. In this research, the NH_2_-FF-COOH peptide configuration of the nanotubes demonstrated the most potent activity against staphylococcal planktonic and biofilm forms of bacteria. These findings were mainly due to formation of ion channels in the bacterial cell membrane and/or due to the surfactant-like action of the nanotubes that allowed them to selectively target the cell membrane and permeate the biofilm matrix. Altogether, these effects resulted in total biofilm eradication for the tested Gram-positive bacterial isolates. Despite not being as efficient against Gram-negative bacteria, the authors suggested that this approach could be combined with other molecules capable of disrupting the outer membrane of Gram-negative biofilm bacteria, such as the glycopeptide vancomycin or the macrolide erythromycin. Considering the possible synergistic effects, this combination could constitute future therapies in the treatment of medical device, bone and wound infections attributed with high rates of treatment failure, and antibiotic resistance due to the presence of biofilms [85].

In a different approach, it was shown that the development of antimicrobial dendritic polymers represents an alternative infection control strategy, as reported in Rozenbaum et al. [86]. In their work, compact dendrons with different peripheral compositions were studied regarding their penetration ability into *P. aeruginosa* biofilms. The results allowed the researchers to conclude that penetration and accumulation of dendrons into biofilms are controlled by their pH-responsive peripheral composition (OH, COO^-^, or NH_3_^+^ groups), through electrostatic double-layer interactions. Moreover, this conclusion allowed for a better understanding and further development of new antimicrobial dendritic polymers or drug nanocarriers [86]. In fact, dendrimers are unique architectural molecules that are highly branched with a well-defined structure, molecular weight, and surface functionality and with low polydispersity, which, all in all, make them an attractive carrier molecule capable of accommodating both hydrophobic and hydrophilic agents [87].

As for the use of liposomes, Ibaraki et al. [88] reported the design of liposomes with different surface properties using ionic lipids, cholesterol, and polyethylene glycol (PEG)-modified lipids. This study allowed the researchers to infer which design should be considered the most useful carrier system for future evaluation of encapsulated antimicrobial agents regarding its contribution to bacterial biofilm damage, permeability, and retention. It was established that cationic and PEG-modified liposomes could be used as effective delivery systems since they were the ones presenting the highest retention as well as permeability properties against *P. aeruginosa* biofilms [88].

### 5.2. Plant-Based Strategies

Plants and their derivatives can act as Ag^+^ ion reducing and capping agents, thereby enabling simple and eco-friendly synthesis of AgNPs, which results in biodegradable and biocompatible drug delivery solutions without the use of toxic chemicals [26]. Nevertheless, medicinal and aromatic plants per se constitute a large part of natural flora and are considered an important resource in various fields, especially in the pharmaceutical, flavor and fragrance, perfumery, and cosmetic industries [89]. According to World Health Organization (WHO), 80% of the global population is dependent on traditional plant-based medications for treating various human health problems [90].

**Figure 5:**
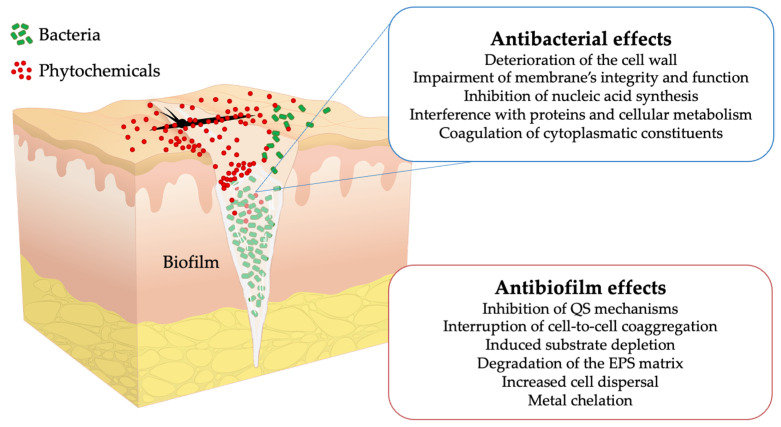
Illustration of an SSI and mode of action of phytochemicals against biofilms and their residing bacterial cells.

Plants contain large amounts of bioactive non-nutrient secondary metabolites, known as phytochemicals, within their leaves, stems, fruits, nuts, and seeds. Added to the increased protection against both biotic and abiotic plant stresses, phytochemicals have been recognized for their antibacterial, antifungal, antiviral, insecticidal, nematicidal, anti-oomycete, antimalarial, antidiabetic, anticancer, antioxidant, anti-inflammatory, antifever, and immunosuppressive properties [91]. In this sense and considering their unique role in the self-defense mechanisms of plants against pathogenic microorganisms, phytochemicals have emerged as a promising alternative to current antimicrobial agents. In addition, phytochemicals can be effective against multidrug-resistant bacteria, including *S. aureus*, *E. coli*, and *K. pneumoniae*, in both planktonic and biofilm forms [92,93]. The strongest antibiofilm properties are attributed to different classes of natural compounds, such as phenolics, essential oils, terpenoids, lectins, alkaloids, polypeptides, and polyacetylenes [94]. These groups of phytochemicals operate on biofilms through several main mechanisms (Figure 5): (1) inhibition of QS mechanisms; (2) impairment of membrane integrity and cell wall degradation; (3) deterioration of the EPS matrix; (4) interference with proteins, DNA, and important cellular reactions; (5) substrate depletion and metal chelation; and (6) interruption of cell-to-cell coaggregation [95]. Moreover, phytochemicals can inhibit the QS mechanism primarily by blocking intercellular communication inducers, thereby suppressing signal transduction; play a significant role in inhibiting bacterial adhesions and suppression of genes involved in biofilm formation, and have the potential to interfere with the biofilm’s access to nutrients essentially required for adhesion and bacterial growth [96].

Since biofilm-associated bacteria are particularly problematic because they can withstand host defenses, antimicrobials, and other stresses more easily than analogous free-living bacteria, some of the presently available synthetic drugs fail to inhibit many multidrug-resistant pathogenic microbes when biofilms are involved. Therefore, exploitation and further development of new, safe, eco-friendly, and efficient antibiofilm strategies and therapeutic approaches are required [90]. Recent studies on this topic have been focusing on assessing the antimicrobial effects of different extracts obtained from plant sources (see Supplementary Materials Table S1). These extracts are rich in phytochemicals likely the same as in plants’ natural states, and these bioactive synergistic compositions provide them with effective defense mechanisms against microorganisms and are less likely to trigger the development of resistance [55]. In particular, Aygül et al. [55] investigated the antibacterial and antiviral effects of *Hypericum lydium* aqueous and ethanolic extracts against *E. coli*, *S. aureus*, and MRSA strains. The results obtained allowed the researchers to verify that the ethanolic extract was able to inhibit the bacterial growth, biofilm formation, and even hemolytic activity of standard strains as well as MRSA clinical isolates, whereas the water extract did not present the same effects. According to the authors, the anti-hemolytic, antibiofilm, and antibacterial activities of *H. lydium* ethanolic extract against *S. aureus* are due to the presence of phytochemicals, such as (−)-epicatechin, quercetin-like, and chlorogenic acid-like compounds. Therefore, the ethanolic extract of this study was considered appropriate to be involved in drug formulations designed for the treatment of MRSA-associated SSIs, as long as further in vivo research and toxicity evaluations are conducted [55]. Galvão et al. [56] addressed the highly prioritized MRSA bacterium, which is currently on the global list of antibiotic-resistant bacteria requiring research and innovative, effective treatment options. In this study, aqueous and ethanolic extracts of *Cochlospermum regium* leaves were obtained to target isolated hospital- and community-acquired MRSA planktonic and biofilm cells. The leaf extract composition revealed elevated concentrations of phenols, as well as gallic and ellagic acids, which when combined promoted membrane permeability changes and decreased enzymatic activity, nutrient and metal ions’ depletion, and interference with genetic regulation of the biofilm formation process [56]. Ekom et al. [57] further explored the antibacterial activity and the wound healing properties of the methanolic extract of the *Persea americana* seed. For that purpose, the extract was tested against some bacterial strains including clinical isolates, both in planktonic and biofilm states. Furthermore, this extract was administered in a gel-based formulation to rat models with wounds infected by *S. aureus* isolates. The *P. americana* seed extract exhibited significant antibiofilm activity, considerably increased the percentage of wound closure, and drastically reduced the colony forming units (CFUs) of *S. aureus* at the infection site, without inducing skin irritation. These antibacterial and antibiofilm properties of the *P. americana* seed are largely attributable to its total phenolic, flavonoid, and tannin contents, which promote severe perturbation of the bacterial membrane, leakage of intracellular materials, and incapacity to regulate crucial cell reactions [57]. A study designed by Okba et al. [37] focused mainly on the chemical composition and virulence inhibition of three common *Iris* species (*I. confusa*, *I. pseudacorus*, and *I. germanica*). In the study, the antibacterial effects of the rhizome and root extracts were tested against four prevalent pathogenic bacteria, namely *E. coli*, *Enterobacter aerogenes*, *Bacillus sphaericus*, and *S. aureus*. The biofilm inhibition and anti-hemolytic activities of the aforementioned *Iris* species were also tested against *S. aureus* bacterial strains. Metabolite profiling of the investigated species allowed the researchers to correlate the detected metabolites with the observed activities, which led to the conclusion that the *I. pseudacorus* extract, of all three, exhibited the strongest antibiofilm and anti-hemolytic effects due to the presence of nigricin and tectorigenin-type isoflavonoids, along with xanthones. These compounds were responsible for causing impairment of the phospholipid biosynthesis of the bacterial cell membrane, as well as interfering with the QS system [37]. Likewise, Jain et al. [58] aimed to determine the antibacterial and antibiofilm activities of alkaloid and flavonoid rhizome extracts obtained from three *Curcuma* species (*C. longa*, *C. caesia*, and *C. aromatica*) against *S. aureus* and *Bacillus subtilis* strains. The results presented in this work suggested that *C. aromatica* flavonoid and alkaloid extracts are a potential source of antibacterial, biofilm dispersal, and antibiofilm agents against Gram-positive bacteria, as compared to the other two *Curcuma* species [58].

Other studies that included plant extracts were performed recently (see Supplementary Materials Table S1). One of those was conducted by Đukanović et al. [38], where the researchers investigated the antibacterial and antibiofilm activities of the *Frangula angus* ethyl-acetate bark extract towards *S. aureus* strains and clinical isolates from surgical wounds, blood, and the nasal carriage. The authors demonstrated the extracts’ influence on cell respiration in both planktonic and biofilm forms. Regarding the composition of the *F. angus* extract, several qualitative and quantitative analyses were performed, which overall allowed the researchers to evidence the presence of phenols, flavonoids, and emodin, whereas catechin and 4-ethoxy benzoic acid were the most prevailing compounds. Moreover, the results obtained in this work showed that *F. angus* possesses strong potentiality since it prevented biofilm formation and disrupted pre-established biofilms in almost all tested strains, suggesting its promising applications in developing new strategies for controlling biofilm growth in nosocomial infections [38]. A study carried by Nadaf et al. [59] explored the antimicrobial, antibiofilm, and also antioxidant activities of *Hymenocallis littoralis* methanolic leaf extracts against pathogenic microorganisms, using experimental and computational biology approaches. In that study, various phytochemicals within the methanolic extract were identified, namely apigenin 7-(4″,6″-diacetylalloside)-4′-alloside, catechin 7-O-apiofuranoside, emodic acid, epicatechin 3-O-*β*-D-glucopyranoside, 4–methylesculetin, methylisoeugenol, quercetin 5,7,3′,4′-tetramethyl ether 3-rutinoside, and 4–methylumbelliferyl *β*-D-glucuronide. It was concluded that the presence of these specific phytochemicals strongly contributed to the antibiofilm properties of the *H. littoralis* extract, by binding at the active sites of residues of adhesin proteins, thereby inhibiting the adhesion process of biofilm formation. Lastly, this phytochemical analysis allowed the researchers to justify the good antioxidant activity of the extract, which is due to the presence of high amounts of phenols and flavonoids [59]. Shehabeldine et al. [39] evaluated the role of methylene chloride-methanol extract of *Callistemon citrinus* and its isolated compounds on *S. aureus* strains. Three major phytochemicals were isolated—pulverulentone A, 8-desmethyl eucalyptin, and eucalyptin—which exhibited significant antibiofilm activity and inhibition of staphyloxanthin biosynthesis, compromised the integrity of bacterial cell membranes, and destroyed the biofilm architecture by reducing its thickness and overall biomass. Therefore, *C. citrinus* phenolics and acylphloroglucinols may serve as a potential source of plant-based antibacterial and antibiofilm agents, as they modulate the QS system, interfere with surface hydrophobicity, mobility, and charge, and downregulate important biofilm formation genes. Besides this, they could be further implicated to control MRSA biofilm-associated diseases. [39].

As previously mentioned in this review, it is well known that most pathogenic bacteria coordinate their complex virulence response mechanisms through a highly structured network of cell-to-cell communication. Many phytochemical isolated compounds and bioactive extracts have already demonstrated the ability to inhibit the QS mechanism [96]. For example, the investigation conducted by Alyousef et al. [40] demonstrated that the methanolic leaf extract of *Myrtus communis* was highly effective at interfering with biofilm formation, EPS production, and swarming motility, as well as inhibiting QS-regulated virulence in uropathogenic strains usually related to biofilm-based persistent infections. In that study, the importance was also underlined of linalool, confirmed as one of the major constituents of the *M. communis* extract, on the observed effects. The linalool mode of action has been correlated with the inhibition of acyl-homoserine lactone signal molecule synthesis, antagonization of QS-regulatory proteins, and blocking of the receptor proteins [40]. Kalia et al. [41] used a single isolated phytochemical, parthenolide, which is a sesquiterpene lactone obtained from the *Tanacetum parthenium* plant, and investigated its anti-QS activity against *P. aeruginosa* bacterium biofilms. They found that parthenolide was able to significantly decrease biofilm formation and hinder virulence factors, showing remarkable downregulation of QS signaling molecules’ synthesis and their respective receptors [41]. In Usmani et al.’s work [42], ursolic acid and its amide derivatives, particularly N-(2′,4′-dinitrophenyl)-3*β*-hydroxyurs-12-en-28-carbonamide), were explored for their antibacterial and antibiofilm potential against the colistin-resistant *A. baumannii* (CRAB) reference and clinical isolate strains. Although the compound did not eradicate the bacterial isolates, it presented a strong anti-virulent and bacteriostatic nature, while inhibiting bacterial growth, rupturing and eradicating biofilm formation, and reversing the resistance mechanism of *A. baumannii* by depolarization of the cell membrane. This synthetic amide derivative of ursolic acid also restrained the expression of several genes, including QS genes encoding receptor proteins and transcriptional regulatory factors [42]. von Borowski et al. [33] considered the urgency of identifying novel antiadhesion agents as an alternative method to prevent bacterial attaching, biofilm formation, and infections including SSIs. For that, the authors investigated *Capsicum baccatum* fruit and seed extracts against *P. aeruginosa* and *S. epidermidis* biofilms using different extraction solvents. The most active extract of *C. baccatum* was then incorporated into a polymeric surface by the spin-coated technique, which allowed for production of a highly hydrophobic, anti-infective modified surface. This positive and indeed interesting outcome evidenced the potential of *C. baccatum* to be used as a source of natural compounds for the development of effective antibiofilm strategies to control clinical and industrial problems associated with microbial contamination [33]. Akhtar et al. [34] studied the applicability of a combination of chitosan, bioactive glass, and ferulic acid, which is a phenolic phytochemical with a wide range of biological activities, resulting in ferulic acid-loaded composite coatings. According to this study’s findings, ferulic acid induced modifications in the bacterial cell membrane, changes in hydrophobicity, and local rupture of the cell membrane with consequent leakage of intracellular bacterial components. In vitro cell culture assays allowed the researchers to confirm that incorporating ferulic acid in the coatings increased the viability of human osteoblast-like cells alongside antibacterial tests, which demonstrated the strong bactericidal activity of the coatings against *E. coli* and *S. aureus* strains. Overall, the combination showed remarkable results for the use of the composite coatings to improve metallic implants already available on the market and prevent their contamination during surgical procedures [34]. Another interesting approach was presented by Jardak et al. [60], who studied the antibacterial and antibiofilm activities of essential oils from four different plant species, namely *Piper nigrum*, *Cuminum cyminum*, *C. longa*, and *Cinnamomum verum*, against several bacterial strains and *S. epidermidis* biofilms. The results obtained highlighted the strong antibiofilm activity of *C. verum* essential oil, and also the effect of the combination comprising the mixture of the four essential oils, which not only reduced the biofilm’s thickness but also strongly decreased its viability at low concentrations. Moreover, phytochemical composition analysis showed that the antibacterial effects of the essential oils might be due to the presence of high contents of eugenol in *C. verum*, *β*-turmerone and *α*-curcumene in *C. longa*, cuminaldehyde in *C. cyminum*, and limonene, *β*-caryophyllene, *α*-pinene, *δ*-3-carene, and *β*-pinene in *P. nigrum* essential oils. These compounds efficiently disrupt and damage the cellular membrane’s integrity due to interactions with the phospholipids of the membrane [60].

Despite intensive research and investigation of phytochemicals as antibiofilm agents under in vitro and in vivo testing, no drug approved by the Food and Drug Administration (FDA) has been developed. This might be due to most of them have failed in clinical trials, as the availability of the compounds in humans after administration tends to decrease. To overcome this problem and advance in the antibiofilm activity, a combination strategy that includes the use of commercial antibiotics alongside phytochemicals needs to be studied [96] (see Supplementary Materials Table S1). As such, Ferreira et al. [61] assessed the antibacterial, antifungal, and antibiofilm effects of a lectin isolated from the *Alpinia purpurata* inflorescence bract extract (ApuL) against human pathogens, including *S. aureus* and *P. aeruginosa* (standard and antibiotic-resistant isolates) as well as *Candida* species. In addition, the research findings confirmed that combining this lectin with common antibiotics, such as ceftazidime and fluconazole, resulted in some synergistic activities against resistant isolates and fungal species. ApuL is an acidic and oligomeric protein that, in this study, showed remarkable results in terms of bacterial and fungal growth inhibition due to impairment of cell viability, confirmed by the evaluation of growth curves, protein leakage, and ultrastructural changes. The antifungal effects of ApuL are mainly due to its ability to bind to chitin, chitin oligomers, cellulose, and other saccharides in the cell walls, inhibiting fungal growth. Also, lectins cause oxidative stress, energetic collapse, and enter fungal cells, thus blocking enzymes involved in the synthesis of wall polymers [61]. Another study that explored the combined effects of phytochemicals obtained from plant sources and conventional antibiotics was developed by Lai et al. [62]. The antibiotics gentamicin, chloramphenicol, penicillin G, vancomycin, and ampicillin were combined with the polyphenol-rich fraction of *Dicranopteris linearis*, and its antibacterial and antibiofilm activities were evaluated against *S. aureus* and *P. aeruginosa*. The authors concluded that some combinations, once again, resulted in synergistic effects, increasing the activity of the used antibiotics, and thereby reversing the bacterial response from resistant to susceptible towards these antimicrobial agents [62]. In Dias-Souza et al.’s work [63], the antibacterial and antibiofilm activities of the methanolic extract of *Euterpe oleracea* fruit were evaluated against clinical isolates of *S. aureus*. Additionally, other antimicrobial drugs were combined with the extract to obtain synergistic interactions, including ciprofloxacin, erythromycin, and chloramphenicol. Despite the need for additional in vivo studies and cytotoxic tests, this work contributes to developing more promising strategies for biofilm eradication [63]. Neto et al. [64] investigated the use of curcumin alone and in combination with oxacillin to eradicate MRSA biofilms, and analyzed through flow cytometry and molecular docking the mechanism responsible for causing cell death. The results showed that curcumin causes changes in membrane integrity and DNA fragmentation, indicating that MRSA cell death might be related to apoptotic processes [64]. Deepika et al. [65] optimized a simple chromatographic method for the isolation of rutin, a vitamin P-rich flavonoid group of phytochemicals with antimicrobial properties, from *Citrus sinensis* peels. The isolated phytochemical was studied as a potential antibiofilm agent against *P. aeruginosa* pathogenic bacterium in combination with antibiotic gentamicin. It was concluded that the rutin-gentamicin combination presented enhanced antibiofilm activity due to ROS generation in bacteria, leading to oxidative stress and death. Consequently, this approach gains significance since rutin can minimize the dose and cost of antibiotics used to control and treat bacterial infections, such as SSIs associated with biofilm-forming pathogenic bacteria [65].

## 6. Implementation of Detection and Real-Time Monitoring Systems to Improve Biofilm Control Strategies

With all the advances and innovation in the field of novel antimicrobial and antibiofilm strategies, some of which having already achieved groundbreaking results, it is still necessary to develop accurate diagnostic tools for the detection and monitoring of bacterial infections. These infections often progress to more complex biofilm-related infections that are hard to detect by simple and non-invasive techniques and are easily mistaken for sterile inflammations. Therefore, in the last few years, new diagnostic tools were invented specifically for clinical environments, providing high-resolution and practical medical procedures for the treatment of bacterial infections [80].

The application of machine learning algorithms has been employed in more recent studies to develop quantitative activity-composition relationship classification models that allow researchers to easily determine which antimicrobial agent (when more than one is involved) has stronger antimicrobial or antibiofilm activities. Thus, these algorithms can help researchers to understand the utility of certain agents in the prevention or eradication of bacterial contamination and to reduce human infections [97]. Moreover, another interesting approach is to combine machine learning with image processing, thereby assisting doctors during clinical and diagnostic processes. As reported in the literature, machine learning systems can be an asset to determine bacterial concentrations in biofilms, while deep learning models have already been applied for detection and characterization of all four stages of biofilm formation [80,98]. Other deep learning models, such as variations of convolutional neural networks, were used to extract the cells’ geometrical properties from microscopy features, allowing researchers to explain how the biofilm structurally adapts to the surface properties [99]. More importantly, these deep learning models based on artificial intelligence can be trained to detect polymicrobial biofilms with 90% accuracy in contrast to 50% when compared to human experts, thus offering an accurate alternative to the commonly used and time-consuming biochemical methods [80,98].

On the other hand, strategies involving ultrasonic imaging have also been explored in order to allow the identification of biofilms in early stages [100,101]. This approach alongside other important improvements of ultrasound contrast agents, such as encapsulated gas micro and nanobubbles, constitutes an advantage for monitoring the formation and growth of biofilms in real-time, as well as establishing the difference between infectious and healthy tissues [80]. Nevertheless, other studies in the literature also indicate that the use of ultrasound-assisted therapies can be efficient not only to detect early and mature biofilms, but also, to some extent, to help combat these microbial communities. For instance, ultrasounds have been reported to enhance antibiotic treatment by improving antibiotic efficacy, increasing cell death, and reducing biofilm thickness [102]; or the use of acoustically activated microbubbles that facilitate physical perturbation of the biofilm and provide the means to control drug delivery both temporally and spatially [103].

The abovementioned approaches demonstrate significant progress in understanding the biological reactions that lead to biofilm formation or even eradication through real-time analysis assisted by microscopic, spectrochemical, electrochemical, and piezoelectrical methods [104]. However, several other approaches that were not referred to in the course of this review, especially ones based on synthetic biology, deserve to be mentioned as these hold substantial promise for controlling biofilms by improving and expanding existing biological tools [105]. These include protein engineering of global regulators or signaling molecule-binding proteins to hinder or modulate biofilm formation [106,107]; QS circuit systems for controlling biofilms [108]; quorum quenching enzymes and chemical compound production systems [109,110]; genetically engineered phages with biofilm inhibitory functions [111,112,113]; and probiotics with synthetic genetic circuits that enable antibiofilm activity [114,115,116,117].

## 7. Concluding Remarks and Challenges

Biofilms pose serious challenges to the global healthcare community as they are responsible for many difficult-to-treat infections, including SSIs, caused by pathogenic multidrug-resistant and biofilm-forming bacteria. Since biofilms are particularly problematic due to their inherited tolerance to host immune defenses, antimicrobials, and other stresses, some of the presently available conventional antibiotics are unable to completely treat infections, triggering the development of resistant bacteria.

In recent years, many researchers have focused their attention on the development of new, safe, environmentally conscious, and efficient antibiofilm strategies as alternatives to conventional approaches. Recent studies regarding NP and phytochemical-based approaches were put together and discussed in this review. Numerous studies showed remarkable results for the prevention of biofilm formation, as well as total eradication of pre-established biofilms of different microorganisms frequently associated with human infections. Moreover, in some cases, NPs and phytochemicals have been explored in regard to restoring the lost antibacterial and antibiofilm efficacy of in-use antibiotics. It was confirmed that these approaches constitute highly promising resistance-modifying antibiofilm agents and potent adjuvants that enhance the activity of conventional antibiotics through synergistic effects obtained in different combinations.

Although various antibiofilm strategies have been developed, it is still necessary to carry out additional studies to overcome issues associated with the lack of mechanistic and biological understanding of compound activity/biofilm interactions. More in vivo studies, alongside the need to standardize the in vitro methodologies, are crucial to evaluate the antimicrobial and antibiofilm activities of the explored agents, as in vitro studies do not always predict in vivo outcomes. Finally, future clinical trials would allow for better comprehension of the biofilm role in SSIs, since real-life environments, e.g., postoperative surgical wound infections, are associated with high microbial diversity, contrasting with laboratory experiments using pure bacterium cultures.

## Figures and Tables

**Table 1:** Recommended antibiotic prophylaxis to prevent SSIs caused by bacterial strains in different types of surgical procedures and modes of action of the different antibiotic classes. Adapted from Salkind et al. [18].

Type of Surgical Procedure	Bacterial Strain	Recommended Antibiotic(s)	Antibiotic Class(es)	Mode of Action
Cardiothoracic	*S. aureus*, coagulase-negative staphylococci	Cefazolin ^1^	Cephalosporins	Disruption of peptidoglycan synthesis
		Cefuroxime ^2^		
Orthopedic		Vancomycin ^3^	Aminoglycosides	Inhibition of protein synthesis
Gastrointestinal	Enteric Gram-negative bacteria, anaerobes, enterococci	Cefoxitin ^4^	Cephalosporins	Disruption of peptidoglycan synthesis
		Cefotetan ^5^		
		Ampicillin ^6^/Sulbactam ^7^	Beta-lactams	
		Cefazolin + Metronidazole ^8^	Cephalosporins + Nitroimidazoles	Disruption of peptidoglycan synthesis + inhibition of protein synthesis and degradation of DNA
Gynecologic (vaginal, abdominal, or laparoscopic hysterectomy)	Enteric Gram-negative bacteria, group B streptococci, enterococci, anaerobes	Cefoxitin	Cephalosporins	Disruption of peptidoglycan synthesis
		Cefotetan		
		Cefazolin		
		Ampicillin/Sulbactam	Beta-lactams	
Vascular	*S. aureus*, coagulase-negative staphylococci, enteric Gram-negative bacilli	Cefazolin	Cephalosporins	Disruption of peptidoglycan synthesis
		Vancomycin	Aminoglycosides	Inhibition of protein synthesis

## Data Availability

Not applicable.

## Supplementary Materials

The following are available online at https://www.mdpi.com/article/10.3390/antibiotics11010069/s1, Table S1: Recent studies on the use of nanoparticle and phytochemical-based approaches to counteract biofilm-related SSIs.
