# Supplementary material for: Biofilms in Surgical Site Infections: Recent Advances and Novel Prevention and Eradication Strategies
Source: Antibiotics (Basel). 2022 Jan 7;11(1):69. doi: 10.3390/antibiotics11010069 (PMC8773207; doi:10.3390/antibiotics11010069)
Supplement: Supplementary file 1 [file antibiotics-11-00069-s001.zip › antibiotics-1477802-supplementary.pdf]

Supplementary Materials

Biofilms in Surgical Site Infections: Recent Advances and Novel Prevention and Eradication Strategies

Andriy Hrynyshyn, Manuel Simões and Anabela Borges

1. Table S1: Recent studies on the use of nanoparticle and phytochemical-based approaches to counteract biofilm-related SSIs.

Table S1. Recent studies on the use of nanoparticle and phytochemical-based approaches to counteract biofilm-related SSIs.

Antibiofilm Strategy			Microorganism(s)	Source(s)	Mechanism(s) of action	Reference
**Approach**	**Compound(s)**	**Method(s)**				
**Nanoparticles**	AgNPs	Coating of silk sutures	*C. albicans*	ATCC 10239	Protein-AgNP interactions, inhibition of DNA replication, disruption of the microbial cell wall and induced formation of ROS	[1]
			E. coli	ATCC 25922		
			S. aureus	ATCC 25923		
	AgNPs	Coating of silk sutures	*S. aureus*	ATCC 25923 + clinical isolate(s)	Attachment of AgNPs to cell membrane and penetration into the cytoplasm, causing cell lysis.	[2]
			A. baumannii	ATCC 19606 + clinical isolate(s)		
			E. coli	ATCC 25922 + clinical isolate(s)		
			K. pneumoniae	ATCC 700603 + clinical isolate(s)		
			P. aeruginosa	ATCC 27853 + clinical isolate(s)		
	TCA, "Cinn" and PI-based AgNPs<br><br>(TCA-AgNPs; TCA-AgNPs-PI; Cinn-AgNPs; and Cinn-AgNPs-PI)	Combination with phytochemicals + coating of PGA sutures	*S. pneumoniae*	ATCC 49619	TCA, in an acidic environment, remains lipophilic and enters the bacterial cell membrane; establishes electrostatic interactions, leading to adsorption, deformation of the membranes and its destruction; it has a phenolic ring which interacts with the proteins in the cytoplasmic membranes and causes leakage of cell constituents; it is oxidizing to AgNPs, causing Ag^{+} ions release, which are detrimental for the cell membranes.<br><br>PI has microbicidal activity due to release of free molecular iodine.<br><br>"Cinn" has biocidal properties and in water forms a high amount of cinnamaldehyde, which is oxidized to cinnamic acid.	[3]
			S. aureus	ATCC 25923		
			E. faecalis	ATCC 29212		
			S. pyogenes	ATCC 19615		
			B. subtilis	Clinical isolate(s)		
			P. aeruginosa	WDCM 00026 Vitroids		
			E. coli	WDCM 00013 Vitroids		
			P. mirabilis	ATCC 29906		
			K. pneumoniae	WDCM 0007 Vitroids		
			C. albicans	WDCM 00054 Vitroids		
	AgNPs	Coating of nylon sutures	*S. aureus*	ATCC 25923	The increased surface area of the AgNPs provides better contact with microorganisms, leading to attachment of NPs to cell membrane, penetration into the cytoplasm and cell lysis.	[4]
			E. coli	ATCC 25922		
			P. aeruginosa	ATCC 27853		
			A. baumannii	ATCC 19606		

			K. pneumoniae	ATCC 700603		
	Ag^+–TiO_2 NPs (TIAB) + *Aloe vera* extract + hyaluronic acid	Coating of braided multifilament sutures	*S. aureus*	ATCC 29213	Ag^+ ions cause bacterial proteins inactivation, inhibition of DNA replication, modulation of gene expression, blockade of the electron transport chain with the consequent reduction of ATP, and ultimately, interference with QS.	[5]
			E. faecalis	ATCC 29212		
			E. coli	ATCC 25922		
	AgNPs	Coating of cotton gauze wound dressings	*E. coli*	ATCC 25922	Ag^+ ions are responsible for the bactericidal activity, by disrupting the vital biomolecules of bacteria (cell membrane, DNA and enzymes).	[6]
			S. aureus	ATCC 6538		
			MRSA	ATCC 33591		
	AgNPs + gentamicin	Coating of a porous structure on a titanium implant surface	*S. aureus*	ATCC 25923	Induced production of ROS.<br><br>Combination of AgNPs and gentamicin creates a synergistic effect.<br><br>Gentamicin targets and attacks the 30S ribosomal subunit.	[7]
	AgNPs + CaPNPs	Coating of Ti6Al4V metallic scaffolds	*S. aureus*	NCTC 657	Ag^+ ions release has antibacterial effects.<br><br>PEI used to stabilize CaPNPs has antibacterial and toxic effects to cells because of its positive charge, which affects cell membranes.	[8]
	AgNPs	Targeted delivery of NPs using DMNs	*S. aureus*	ATCC 25923	Disturbed membrane permeability, leading to its disruption, leakage of intracellular content, damage to DNA and death of bacterial cells.<br><br>Incorporation of AgNPs into microparticles of PCL coated with chitosan and delivery through DMNs reduces toxicity and enhances antibiofilm activity at the exact area of infection.	[9]
			P. aeruginosa	ATCC 9027		
	PLGA-NPs and PCL-NPs	Targeted delivery of NPs using doxycycline DMNs	*S. aureus*	NCTC 10788 + ATCC BAA-1707	PLGA and PCL coated with chitosan NPs improve adhesion to bacterial biofilms.<br><br>Doxycycline has bactericidal effects.<br><br>DMNs allow better penetration at the targeted area of infection.	[10]
			P. aeruginosa	ATCC 9027 + ATCC BAA-47		
	CAR PCL-NPs	Combination with phytochemicals +	*S. aureus*	NCTC 10788 + ATCC BAA-	CAR interacts with cell membrane and promotes loss of integrity (hydrophobic nature).	[11]

		targeted delivery of NPs using microneedle liquid injection system		1707 + ATCC 33593	CAR’s slightly hydrophilic side enhances its diffusion through the biofilm EPS matrix.<br><br>Encapsulation of CAR in NPs improves its antimicrobial activity.<br><br>Microneedle liquid injection system improves site-specific delivery of CAR-PCL NPs.	
			P. aeruginosa	ATCC 9027 + ATCC BAA-47		
	CAR PCL-NPs	Combination with phytochemicals + targeted delivery of NPs using a hydrogel system	MRSA	ATCC 33593 + ATCC BAA-1707	CAR disrupts proton gradients and inhibits adenosine triphosphate (ATP) synthesis.<br><br>CAR hinders efflux pumps, produces ROS, suppresses enterotoxin and coagulase virulence factors and, overall, inhibits QS.<br><br>CAR-loaded PCL-NPs prolong the availability of CAR in skin layers, thereby improving its antibacterial activity.<br><br>Loading NPs into the hydrogel increases the retaining of NPs in the skin for longer durations.	[12]
	Photothermal PDA-NPs	NIR irradiation of photothermal NPs	*E. faecium* W54	–	Photothermal NPs convert NIR-light into heat and generate high, local temperatures that imply lethal damage to cell wall components, eDNA and other intra- or extra-cellular material.<br><br>NIR-irradiation can be confined to the infection site and heat helps to dissipate NPs in the biofilm.	[13]
			S. aureus	ATCC 12600		
			K. pneumoniae	–		
			A. baumannii	–		
			P. aeruginosa PA01	–		
			E. cloacae BS1037	–		
			E. faecalis 1396	–		
	D/L-Glu AuNBPs	*In vivo* injection of NPs + NIR-irradiation	*S. epidermidis*	–	D/L-Glu functionalization enhances the targeting and interactions of AuNBPs with bacterial cell walls leading to biological restriction of nucleic acids.<br><br>The sharp tips and small size of AuNBPs enable the penetration into bacterial cell wall and	[14]
			E. coli	–		

					biofilms and cause damage and leakage of components.<br><br>The targeting to bacterial wall enhances photothermal therapy against biofilm infections.	
	AuNPs + gentamicin or amikacin	Targeted laser therapy + antibiotics	MRSA SA5120	Clinical isolate(s)	AuNPs-targeted laser therapy induces photothermal destruction of the EPS matrix and cellular components of the biofilm and potentiates the antibiofilm activity of gentamicin and amikacin.	[15]
			P. aeruginosa PA 60–65	Clinical isolate(s)		
	MNPS-NPs	Intravenous injection of magnetic NPs + magnetic implant	*S. aureus* JSNZ wildtype	–	MNPS-NPs contain a superparamagnetic iron oxide core, allowing for targeted accumulation of NPs in the infection site.<br><br>The nanoporous silica shell of the NPs can carry large amounts of drug.	[16]
	ZnO-NPs	Multilayer coating of PVC bed sheet	*S. aureus*	DMST 8013	ZnO-NPs release ROS and Zn^{2+} ions which reduce the activity of Zn^{2+}–dependent enzymes and transcription factors and/or cause lysosomal destabilization in the cell, leading to its death.<br><br>ZnO-NPs produce H_2O_2 under UV light irradiation that has toxic effects to living cells.<br><br>ZnO-NPs increases hydrophobicity of the coated material and provides good antibacterial activity.	[17]
			S. epidermidis	DMST 15505		
			E. coli	DMST 4212		
			P. aeruginosa	DMST 4739		
	PCL-NPs + *Thymus capitatus* and *Origanum vulgare* essential oils	Combination with phytochemicals + nanoencapsulation	*S. aureus*	CCM 4223	Encapsulation reduces the loss of bioactivity of the phytochemicals, improves antibacterial and antibiofilm effects, and reduces cytotoxicity to host cells.	[18]
			E. coli	CCM 3988		
			C. albicans SC 5314	–		
	PDA colloidal particles coated internally and externally with AgNPs	Protective sandwich-structured shells coated with NPs	*E. coli*	ATCC 25922	AgNPs deactivate bacterial cells by interacting with sulfide-groups within enzymes and proteins and cause structural changes and functional damage to the cell membrane, leading to cell death.	[19]
			S. aureus	ATCC 29213		

	(AgNPs-PDA-AgNPs shells)				The enhanced antibacterial activity is due to the unique sandwich structure of the samples, in which the external AgNPs offer a rapid and intense release of Ag^{+} ions because of the direct contact to the surroundings, while the internal AgNPs provide a slow yet sustained release of Ag^{+} ions due to the obstruction of the PDA shell.	
	PAA IO-NPs	Polymer capped NPs	*E. coli*	ATCC 25922	The carboxyl groups on the surface of PAA IO-NPs allows the localized deposition of the NPs on the biofilms, which exhibit bioactivity under a low pH environment. This leads to the production of free radicals, which degrade the EPS structure, dispersing the biofilms and killing bacteria embedded within biofilms.	[20]
			S. aureus	ATCC 6538		
			MRSA	ATCC 33592		
	NH_2-FF-COOH nanotubes	–	*S. aureus*	NCTC 10788	Formation of ion channels and/or surfactant-like action, capable of degrading the biofilm matrix and disrupting cell membranes, leading to cell death in Gram-positive bacterial isolates.	[21]
			E. coli	ATCC 15597		
			S. epidermidis	ATCC 12228		
			P. aeruginosa	ATCC 15692		
	Dendrons with OH, COO^{-} and NH_3^{+} peripheral groups	Penetration into biofilm layers + drug-carrier dendritic polymers	*P. aeruginosa*	ATCC 39324	The OH groups remain uncharged inside P. aeruginosa biofilms. EPS components and bacterial cell surfaces, remain negatively charged around pH 6.5.<br><br>The accumulation of NH_3^{+} dendrons near the top of the biofilm is due to strong, electrostatic double-layer mediated adhesion of dendrons, hindering their penetration to deeper layers. The OH and negatively charged COO^{-} dendrons migrate deeper into the biofilms, since they experience no electrostatic double-layer attraction with the channel walls and weak Lifshitz-van der Waals attraction.	[22]
	Ionic and PEG-modified liposomes	–	*P. aeruginosa* PAO-1	JCM 14847	–	[23]
**Phytochemicals**	*Hypericum lydium* extract	–	*E. coli*	ATCC 25922	–	[24]
			S. aureus	ATCC 29213		

			MRSA	Clinical isolate(s)		
	Cochlospermum regium leaf extract	–	*S. aureus*	ATCC 25923	Phenolic compounds of the extract cause changes in membrane permeability, decreased enzymatic activity and depletion of substrates and metal ions.<br><br>Gallic acid interferes with the genetic regulation of the biofilm formation process, reducing the production of EPS.	[25]
			MRSA	Clinical isolate(s)		
	Persea americana seed extract	Topical application of gel-based formulations	*S. aureus*	ATCC 25923 and clinical isolate(s)	Perturbation of the cell membrane, leakage of intracellular liquid, incapacity of the membrane to regulate the internal pH and inactivate the respiratory chain of dehydrogenase.	[26]
			E. coli	ATCC 8739 and clinical isolate(s)		
			P. aeruginosa PA01	–		
	Iris species (*I. confusa*, *I. pseudacorus* and *I. germanica*) rhizome and root extracts	–	*S. aureus*	MTCC 87, ATCC 29213 and clinical isolate(s)	*I. germanica* extract causes impairment in phospholipid biosynthesis of the bacterial cell membrane.<br><br>Interference with the QS system, inactivation of bacterial adhesins and enzymes, altering the cell-substratum interactions, cell membrane, and adherence phase.	[27]
			B. sphaericus	MTCC 511		
			E. coli	MTCC 443		
			E. aerogenes	MTCC 111		
	Curcuma species (*C. longa*, *C. caesia* and *C. aromatica*) flavonoid and alkaloid extracts	–	*S. aureus*	MTCC 96	Curcumin suppresses *B. subtillis* cytokinesis through induction of filamentation, segregation and organization of the nucleoids.<br><br>Curcumin reduces biofilm initiation genes, inhibits QS genes and down-regulates different virulence factors.	[28]
			B. subtilis	MTCC 441		
	Frangula alnus bark extract	–	*S. aureus*	ATCC 25923 and clinical isolate(s)	Inhibition of biofilm formation through the blockade of cell respiration.<br><br>Disturbance of cell membrane and cell lysis.	[29]
			MRSA	ATCC 43300 and clinical isolate(s)		
		–	*C. albicans*	NCIM 3466		[30]

	Hymenocallis littoralis leaf extract				The identified phytochemicals in the extract bind at the active site residues of adhesin proteins, sortase A and Als3, and inhibits substrate binding, preventing the adhesion process of biofilm formation.<br><br>The phytochemicals antioxidant activity helps to reduce excessive ROS generation by the wounded cells, improving wound healing.	
			S. aureus	NCIM 2654		
			B. subtilis	NCIM 2653		
			S. typhimurium	NCIM 2501		
			P. vulgaris	NCIM 2813		
	Callistemon citrinus extract	–	*S. aureus*	ATCC 25923	Phenolic compounds of the extract have intrinsic acidic characteristics which generate a lethal environment against bacteria.<br><br>Phenolic compounds modulate QS, interfere with surface hydrophobicity, mobility and charge, and down-regulate biofilm formation genes.<br><br>Damage to bacterial cell membranes and leakage of cytoplasm constituents.	[31]
			MRSA	ATCC 33591		
	Myrtus communis leaf extract	–	*C. violaceum*	ATCC 12472	Reduced proteolytic lytic (total protease) activity and decreased production of QS controlled virulence determinants in *C. violaceum* (violacein), *P. aeruginosa* (elastase, protease, pyocyanin and chitinase) and *S. marcescens* (prodigiosin and protease).<br><br>EPS production is controlled by bacterial QS mechanism. Therefore, an impaired EPS secretion leads to structurally fragile and antibiotic sensitive biofilm, facilitating its eradication.<br><br>The flagellar mediated swarming motility requires QS-controlled differentiation of normal bacterial cells into swarm cell morphology. However, the phytochemicals of the extract interfere with either the differentiation process	[32]
			E. coli	ATCC 35218		
			P. aeruginosa PAO1	–		
			S. marcescens	ATCC 13880		
			A. baumanii	ATCC BAA-7		

					or act directly on the receptors involved in the QS system.<br><br>Linalool causes inhibition of acyl-homoserine lactone signal molecule synthesis, antagonization of QS-regulatory proteins, and blockade of the receptor proteins.	
	Parthenolide	–	*P. aeruginosa* PAO1	–	Parthenolide has affinity towards the regulatory proteins of the QS circuit, resulting in down-regulation of various virulence factors, such as pyocyanin, protease and swarming motility, thereby significantly reducing biofilm formation.	[33]
	Ursolic acid and its amide derivates,<br><br>N-(2′,4′-dinitrophenyl)-3β-hydroxyurs-12-en-28-carbonamide)	–	*A. baumannii*	ATCC 19606 and clinical isolate(s)	Down-regulation in gene expression of biofilm-forming proteins that engage in cell adhesion, as well as QS regulator genes.<br><br>Powerful membrane disruptor agent, causes depolarization of the bacterial cell membrane, resulting in loss of cytoplasmatic content and cell death.	[34]
	Capsicum baccatum fruit and seed extract	Coating of polymeric surfaces	*P. aeruginosa* PA14	–	The coating inhibits bacterial attachment by a mechanism based on electrostatic repulsion, high hydrophilicity and steric hindrance that blocks bacterium-substratum interactions.	[35]
			S. epidermidis	ATCC 35984		
	Ferulic acid + chitosan + bioactive glass	Coating of metallic surfaces	*S. aureus*	–	Ferulic acid induces modifications in the bacterial cell membrane, changes in hydrophobicity and local rupture of cell membrane with consequent leakage of intracellular bacterial components.<br><br>The combination of Chitosan/bioactive glass with ferulic acid causes changes in bacterial permeability, lysis and subsequently death of bacterial cells.	[36]
			E. coli	–		
	Piper nigrum, *Cuminum cyminum*, *C.longa* and *Cinnamomum*	Combination of essential oils (synergy between phytochemicals)	*B. subtilis*	ATCC 6633	Essential oils disrupt and damage the cellular membrane integrity due to interactions between hydrophobic phytochemicals and phospholipids of the membrane.	[37]
			S. aureus	ATCC 9144		
			S. epidermidis S61	–		
			M. luteus	–		

	verum essential oils		*L. monocytogenes*	–	Eugenol present in *C. verum* essential oil has strong antibacterial activity due to the methyl group in the γ position and double bond in α, β positions of the side chain.<br><br>The overall antibacterial activity of the combination results from the synergistic effect between molecules.	
			P. savastonoi	–		
			E. coli	ATCC 10536		
			S. enterica CIP 80.39	–		
			Erwinia sp.	–		
	Lectin from *Alpinia purpurata* inflorescence bract extract (ApuL) + oxacillin, ceftazidime, or fluconazole	Combination with antibiotics	*P. aeruginosa*	UFPEDA-416, UFPEDA-261 and UFPEDA-262	Impairment of microorganism cells viability.<br><br>Lectins interact with microbial cell wall components such as teichoic and teicuronic acids, peptidoglycans and lipopolysaccharides, triggering growth inhibition, damage to cellular integrity, alteration of membrane permeability and nutrient uptake, induction of oxidative stress, and damage to respiratory processes.<br><br>Lectins bind to chitin, chitin oligomers, cellulose and other saccharides in the cell walls, inhibiting fungal growth. Also, lectins cause oxidative stress and energetic collapse and enter fungal cells, blocking enzymes involved in synthesis of wall polymers.<br><br>Synergism was detected for ApuL-ceftazidime and ApuL-fluconazole.	[38]
			S. aureus	UFPEDA-02, UFPEDA-670, UFPEDA-671 and UFPEDA-672		
			C. albicans	URM 5901		
			C. parapsilosis	URM 6345		
	Polyphenol-rich fraction of *Dicranopteris linearis* + gentamicin, chloramphenicol, penicillin G, vancomycin, or ampicillin	Combination with antibiotics	*S. aureus*	ATCC 29213	–	[39]
			P. aeruginosa	ATCC 27853		
			MRSA	ATCC 43300		

	Euterpe oleracea fruit extract + ciprofloxacin, erythromycin, or chloramphenicol	Combination with antibiotics	*S. aureus*	Clinical isolate(s)	Flavonoids present in the extract cause membrane rupture, inhibition of nucleic acid synthesis, inhibition of DNA gyrase, and hampering cytoplasmic membrane functions and oxygen consumption.	[40]
	Curcumin + oxacillin	Combination with antibiotics	*S. aureus*	ATCC 6538P	Curcumin increases permeability of bacterial membrane, impairs cell metabolism and causes bacterial death.<br><br>Curcumin causes damage to the genetic material, i.e., DNA fragmentation, which together with changes in membrane integrity leads to cell death by apoptotic processes.	[41]
			MRSA	Clinical isolate(s)		
	Rutin (vitamin P) + gentamicin	Combination with antibiotics	*P. aeruginosa*	MTCC 2488	Rutin, gentamicin and combination of rutin-gentamicin induce cell wall disruption, increase cell membrane permeability and reduce cell density.<br><br>Rutin acts as pro-oxidant, induces the generation of ROS that damage the cell membrane, DNA and proteins due to oxidative stress, leading to cell death.	[42]

Notes: NPs – nanoparticles; AgNPs – silver nanoparticles; DNA – deoxyribonucleic acid; ROS – reactive oxygen species; TCA – *trans*-cinnamic acid; “Cinn” – natural cinnamon bark extract; PI – povidone-iodine; PGA – polyglycolic acid; TIAB – molecular system incorporating Ag^+ ions combined with TiO_2; ATP – adenosine triphosphate; QS – quorum sensing; MRSA – methicillin resistant *Staphylococcus aureus*; CaPNPs – calcium phosphate nanoparticles; Ti6Al4V – titanium, aluminum and vanadium alloy scaffold; PEI – poly-ethylenimine; DMNs – dissolving microneedles; PCL – poly(Ɛ-caprolactone); PLGA – poly(lactic-co-glycolic acid); CAR – carvacrol; EPS – extracellular polymeric substance; PDA – polydopamine; NIR – near-infrared; eDNA – environmental DNA; D/L-Glu – chiral glutamic acid; AuNBPs – gold nanobipyramids; AuNPs – gold nanoparticles; MNPS-NPs – magnetic nanoporous silica nanoparticles; ZnO-NPs – zinc oxide nanoparticles; PVC – poly(vinyl chloride); UV – ultraviolet; PAA IO-NPs – poly(acrylic acid) capped iron oxide (IO) nanoparticles; NH_2-FF-COOH – diphenylalanine (FF) peptide nanotubes with amino (-NH_2) and carboxylic acid (-COOH) terminal functional groups; PEG – polyethylene glycol. Microorganism sources: ATCC – American Type Culture Collection; CCM – Czech Collection of Microorganisms; DMST – Department of Medical Sciences Thailand; MTCC – Microbial Type Culture Collection; NCIM – National Collection of Industrial Microorganisms; NCTC – National Collection of Type Cultures; JCM – Japan Collection of Microorganisms; UFPEDA – Collection of microorganism cultures from the Antibiotics Department of the Federal University of Pernambuco; URM – “University Recife Mycologia”, URM Mycotheca Culture Collection of the Department of Mycology of the Center for Biological Sciences of the Federal University of Pernambuco; WDCM – World Data Center for Microorganisms.

References

1. Baygar, T.; Sarac, N.; Ugur, A.; Karaca, I.R. Antimicrobial Characteristics and Biocompatibility of the Surgical Sutures Coated with Biosynthesized Silver Nanoparticles. *Bioorgan. Chem.* **2019**, *86*, 254–258, https://doi.org/10.1016/j.bioorg.2018.12.034.
2. Syukri, D.M.; Nwabor, O.F.; Singh, S.; Ontong, J.C.; Wunnoo, S.; Paosen, S.; Munah, S.; Voravuthikunchai, S.P. Antibacterial-Coated Silk Surgical Sutures by Ex Situ Deposition of Silver Nanoparticles Synthesized with *Eucalyptus camaldulensis* Eradicates Infections. *J. Microbiol. Methods* **2020**, *174*, 105955, https://doi.org/10.1016/j.mimet.2020.105955.
3. Edis, Z.; Haj Bloukh, S.; Ibrahim, M.R.; Abu Sara, H. "Smart" Antimicrobial Nanocomplexes with Potential to Decrease Surgical Site Infections (SSI). *Pharmaceutics* **2020**, *12*, 361, https://doi.org/10.3390/pharmaceutics12040361.
4. Syukri, D.M.; Nwabor, O.F.; Singh, S.; Voravuthikunchai, S.P. Antibacterial Functionalization of Nylon Monofilament Surgical Sutures through In Situ Deposition of Biogenic Silver Nanoparticles. *Surf. Coat. Technol.* **2021**, *413*, 127090, https://doi.org/10.1016/j.surfcoat.2021.127090.
5. Puca, V.; Traini, T.; Guarnieri, S.; Carradori, S.; Sisto, F.; Macchione, N.; Muraro, R.; Mincione, G.; Grande, R. The Antibiofilm Effect of a Medical Device Containing TIAB on Microorganisms Associated with Surgical Site Infection. *Molecules* **2019**, *24*, 2280, https://doi.org/10.3390/molecules24122280.
6. Xiang, J.; Zhu, R.; Lang, S.; Yan, H.; Liu, G.; Peng, B. Mussel-Inspired Immobilization of *Zwitterionic* Silver Nanoparticles toward Antibacterial Cotton Gauze for Promoting Wound Healing. *Chem. Eng. J.* **2021**, *409*, 128291, https://doi.org/10.1016/j.cej.2020.128291.
7. Ständert, V.; Borcherding, K.; Bormann, N.; Schmidmaier, G.; Grunwald, I.; Wildemann, B. Antibiotic-Loaded Amphora-Shaped Pores on a Titanium Implant Surface Enhance Osteointegration and Prevent Infections. *Bioact. Mater.* **2021**, *6*, 2331–2345, https://doi.org/10.1016/j.bioactmat.2021.01.012.
8. Surmeneva, M.; Lapanje, A.; Chudinova, E.; Ivanova, A.; Koptyug, A.; Loza, K.; Prymak, O.; Epple, M.; Ennen-Roth, F.; Ulbricht, M.; et al. Decreased Bacterial Colonization of Additively Manufactured Ti6Al4V Metallic Scaffolds with Immobilized Silver and Calcium Phosphate Nanoparticles. *Appl. Surf. Sci.* **2019**, *480*, 822–829, https://doi.org/10.1016/j.apsusc.2019.03.003.
9. Permana, A.D.; Anjani, Q.K.; Sartini; Utomo, E.; Volpe-Zanutto, F.; Paredes, A.J.; Evary, Y.M.; Mardikasari, S.A.; Pratama, M.R.; Tuany, I.N.; et al. Selective Delivery of Silver Nanoparticles for Improved Treatment of Biofilm Skin Infection Using Bacteria-Responsive Microparticles Loaded into Dissolving Microneedles. *Mater. Sci. Eng. C* **2021**, *120*, 111786, https://doi.org/10.1016/j.msec.2020.111786.
10. Permana, A.D.; Mir, M.; Utomo, E.; Donnelly, R.F. Bacterially Sensitive Nanoparticle-Based Dissolving Microneedles of Doxycycline for Enhanced Treatment of Bacterial Biofilm Skin Infection: A Proof of Concept Study. *Int. J. Pharm. X* **2020**, *2*, 100047, https://doi.org/10.1016/j.ijpx.2020.100047.
11. Mir, M.; Permana, A.D.; Tekko, I.A.; McCarthy, H.O.; Ahmed, N.; Rehman, A.; Donnelly, R.F. Microneedle Liquid Injection System Assisted Delivery of Infection Responsive Nanoparticles: A Promising Approach for Enhanced Site-Specific Delivery of Carvacrol against Polymicrobial Biofilms-Infected Wounds. *Int. J. Pharm.* **2020**, *587*, 119643, https://doi.org/10.1016/j.ijpharm.2020.119643.
12. Mir, M.; Ahmed, N.; Permana, A.D.; Rodgers, A.M.; Donnelly, R.F.; Rehman, A. Enhancement in Site-Specific Delivery of Carvacrol against Methicillin Resistant *Staphylococcus aureus* Induced Skin Infections Using Enzyme Responsive Nanoparticles: A Proof of Concept Study. *Pharmaceutics* **2019**, *11*, 606, https://doi.org/10.3390/pharmaceutics11110606.
13. Gao, R.; van der Mei, H.C.; Ren, Y.; Chen, H.; Chen, G.; Busscher, H.J.; Peterson, B.W. Thermo-Resistance of ESKAPE-Panel Pathogens, Eradication and Growth Prevention of an Infectious Biofilm by Photothermal, Polydopamine-Nanoparticles In Vitro. *Nanomed. Nanotechnol. Biol. Med.* **2021**, *32*, 102324, https://doi.org/10.1016/j.nano.2020.102324.
14. Zhang, M.; Zhang, H.; Feng, J.; Zhou, Y.; Wang, B. Synergistic Chemotherapy, Physiotherapy and Photothermal Therapy against Bacterial and Biofilms Infections through Construction of Chiral Glutamic Acid Functionalized Gold Nanobipyramids. *Chem. Eng. J.* **2020**, *393*, 124778, https://doi.org/10.1016/j.cej.2020.124778.
15. Kirui, D.K.; Weber, G.; Talackine, J.; Millenbaugh, N.J. Targeted Laser Therapy Synergistically Enhances Efficacy of Antibiotics against Multi-Drug Resistant *Staphylococcus aureus* and *Pseudomonas aeruginosa* Biofilms. *Nanomed. Nanotechnol. Biol. Med.* **2019**, *20*, 102018, https://doi.org/10.1016/j.nano.2019.102018.
16. Reifenrath, J.; Janßen, H.C.; Warwas, D.P.; Kietzmann, M.; Behrens, P.; Willbold, E.; Fedchenko, M.; Angrisani, N. Implant-Based Direction of Magnetic Nanoporous Silica Nanoparticles—Influence of Macrophage Depletion and Infection. *Nanomed. Nanotechnol. Biol. Med.* **2020**, *30*, 102289, https://doi.org/10.1016/j.nano.2020.102289.
17. Kurniawan, F.H.; Chinavinijkul, P.; Nasongkla, N. Hydrophobic and Antibacterial Bed Sheet Using ZnO Nanoparticles: A Large-Scale Technique. *J. Drug Deliv. Sci. Technol.* **2021**, *62*, 102339, https://doi.org/10.1016/j.jddst.2021.102339.
18. Kapustová, M.; Puškárová, A.; Bučková, M.; Granata, G.; Napoli, E.; Annušová, A.; Mesárošová, M.; Kozics, K.; Pangallo, D.; Geraci, C. Biofilm Inhibition by Biocompatible Poly(ε-Caprolactone) Nanocapsules Loaded with Essential Oils and Their Cyto/Genotoxicity to Human Keratinocyte Cell Line. *Int. J. Pharm.* **2021**, *606*, 120846, https://doi.org/10.1016/j.ijpharm.2021.120846.
19. Shang, B.; Xu, M.; Zhi, Z.; Xi, Y.; Wang, Y.; Peng, B.; Li, P.; Deng, Z. Synthesis of Sandwich-Structured Silver@Polydopamine@Silver Shells with Enhanced Antibacterial Activities. *J. Colloid Interface Sci.* **2020**, *558*, 47–54, https://doi.org/10.1016/j.jcis.2019.09.091.
20. Nie, L.; Chang, P.; Ji, C.; Zhang, F.; Zhou, Q.; Sun, M.; Sun, Y.; Politis, C.; Shavandi, A. Poly(Acrylic Acid) Capped Iron Oxide Nanoparticles via Ligand Exchange with Antibacterial Properties for Biofilm Applications. *Colloids Surf. B Biointerfaces* **2021**, *197*, 111385, https://doi.org/10.1016/j.colsurfb.2020.111385.
21. Porter, S.L.; Coulter, S.M.; Pentlavalli, S.; Thompson, T.P.; Laverty, G. Self-Assembling Diphenylalanine Peptide Nanotubes Selectively Eradicate Bacterial Biofilm Infection. *Acta Biomater.* **2018**, *77*, 96–105, https://doi.org/10.1016/j.actbio.2018.07.033.
22. Rozenbaum, R.T.; Andrén, O.C.J.; van der Mei, H.C.; Woudstra, W.; Busscher, H.J.; Malkoch, M.; Sharma, P.K. Penetration and Accumulation of Dendrons with Different Peripheral Composition in *Pseudomonas aeruginosa* Biofilms. *Nano Lett.* **2019**, *19*, 4327–4333, https://doi.org/10.1021/acs.nanolett.9b00838.

23. Ibaraki, H.; Kanazawa, T.; Chien, W.-Y.; Nakaminami, H.; Aoki, M.; Ozawa, K.; Kaneko, H.; Takashima, Y.; Noguchi, N.; Seta, Y. The Effects of Surface Properties of Liposomes on Their Activity against *Pseudomonas aeruginosa* PAO-1 Biofilm. *J. Drug Deliv. Sci. Technol.* **2020**, *57*, 101754, https://doi.org/10.1016/j.jddst.2020.101754.
24. Aygül, A.; Şerbetçi, T. The Antibacterial and Antivirulent Potential of *Hypericum lydium* against *Staphylococcus aureus*: Inhibition of Growth, Biofilm Formation, and Hemolytic Activity. *Eur. J. Integr. Med.* **2020**, *35*, 101061, https://doi.org/10.1016/j.eujim.2020.101061.
25. Galvão, F.O.; Dantas, F.G.S.; Santos, C.R.L.; Marchioro, S.B.; Cardoso, C.A.L.; Wender, H.; Sangalli, A.; Almeida-Apolonio, A.A.; Oliveira, K.M.P. *Cochlospermum regium* (Schrank) Pilger Leaf Extract Inhibit Methicillin-Resistant *Staphylococcus aureus* Biofilm Formation. *J. Ethnopharmacol.* **2020**, *261*, 113167, https://doi.org/10.1016/j.jep.2020.113167.
26. Ekom, S.E.; Tamokou, J.-D.-D.; Kuete, V. Methanol Extract from the Seeds of *Persea americana* Displays Antibacterial and Wound Healing Activities in Rat Model. *J. Ethnopharmacol.* **2022**, *282*, 114573, https://doi.org/10.1016/j.jep.2021.114573.
27. Okba, M.M.; Abdel Baki, P.M.; Abu-Elghait, M.; Shehabeldine, A.M.; El-Sherei, M.M.; Khaleel, A.E.; Salem, M.A. UPLC-ESI-MS/MS Profiling of the Underground Parts of Common *Iris* Species in Relation to Their Anti-Virulence Activities against *Staphylococcus aureus*. *J. Ethnopharmacol.* **2022**, *282*, 114658, https://doi.org/10.1016/j.jep.2021.114658.
28. Jain, A.; Parihar, D.K. Antibacterial, Biofilm Dispersal and Antibiofilm Potential of Alkaloids and Flavonoids of *Curcuma*. *Biocatal. Agric. Biotechnol.* **2018**, *16*, 677–682, https://doi.org/10.1016/j.bcab.2018.09.023.
29. Đukanović, S.; Cvetković, S.; Lončarević, B.; Lješević, M.; Nikolić, B.; Simin, N.; Bekvalac, K.; Kekić, D.; Mitić-Ćulafić, D. Antistaphylococcal and Biofilm Inhibitory Activities of *Frangula alnus* Bark Ethyl-Acetate Extract. *Ind. Crop. Prod.* **2020**, *158*, 113013, https://doi.org/10.1016/j.indcrop.2020.113013.
30. Nadaf, N.H.; Parulekar, R.S.; Patil, R.S.; Gade, T.K.; Momin, A.A.; Waghmare, S.R.; Dhanavade, M.J.; Arvindekar, A.U.; Sonawane, K.D. Biofilm Inhibition Mechanism from Extract of *Hymenocallis littoralis* Leaves. *J. Ethnopharmacol.* **2018**, *222*, 121–132, https://doi.org/10.1016/j.jep.2018.04.031.
31. Shehabeldine, A.M.; Ashour, R.M.; Okba, M.M.; Saber, F.R. *Callistemon citrinus* Bioactive Metabolites as New Inhibitors of Methicillin-Resistant *Staphylococcus aureus* Biofilm Formation. *J. Ethnopharmacol.* **2020**, *254*, 112669, https://doi.org/10.1016/j.jep.2020.112669.
32. Alyousef, A.A.; Mabood Husain, F.; Arshad, M.; Rizwan Ahamad, S.; Shavez Khan, M.; Abul Qais, F.; Khan, A.; Alqasim, A.; Almutairi, N.; Ahmad, I.; et al. *Myrtus communis* and Its Bioactive Phytoconstituent, Linalool, Interferes with Quorum Sensing Regulated Virulence Functions and Biofilm of Uropathogenic Bacteria: In Vitro and In Silico Insights. *J. King Saud Univ. Sci.* **2021**, *33*, 101588, https://doi.org/10.1016/j.jksus.2021.101588.
33. Kalia, M.; Yadav, V.K.; Singh, P.K.; Sharma, D.; Narvi, S.S.; Agarwal, V. Exploring the Impact of Parthenolide as Anti-Quorum Sensing and Anti-Biofilm Agent against *Pseudomonas aeruginosa*. *Life Sci.* **2018**, *199*, 96–103, https://doi.org/10.1016/j.lfs.2018.03.013.
34. Usmani, Y.; Ahmed, A.; Faizi, S.; Versiani, M.A.; Shamshad, S.; Khan, S.; Simjee, S.U. Antimicrobial and Biofilm Inhibiting Potential of an Amide Derivative [N-(2′, 4′-Dinitrophenyl)-3*β*-Hydroxyurs-12-En-28-Carbonamide] of Ursolic Acid by Modulating Membrane Potential and Quorum Sensing against Colistin Resistant *Acinetobacter baumannii*. *Microb. Pathog.* **2021**, *157*, 104997, https://doi.org/10.1016/j.micpath.2021.104997.
35. von Borowski, R.G.; Zimmer, K.R.; Leonardi, B.F.; Trentin, D.S.; Silva, R.C.; de Barros, M.P.; Macedo, A.J.; Gnoatto, S.C.B.; Gosmann, G.; Zimmer, A.R. Red Pepper *Capsicum baccatum*: Source of Antiadhesive and Antibiofilm Compounds against Nosocomial Bacteria. *Ind. Crop. Prod.* **2019**, *127*, 148–157, https://doi.org/10.1016/j.indcrop.2018.10.011.
36. Akhtar, M.A.; Mariotti, C.E.; Conti, B.; Boccaccini, A.R. Electrophoretic Deposition of Ferulic Acid Loaded Bioactive Glass/Chitosan as Antibacterial and Bioactive Composite Coatings. *Surf. Coat. Technol.* **2021**, *405*, 126657, https://doi.org/10.1016/j.surfcoat.2020.126657.
37. Jardak, M.; Mnif, S.; Ben Ayed, R.; Rezgui, F.; Aifa, S. Chemical Composition, Antibiofilm Activities of Tunisian Spices Essential Oils and Combinatorial Effect against *Staphylococcus epidermidis* Biofilm. *LWT* **2021**, *140*, 110691, https://doi.org/10.1016/j.lwt.2020.110691.
38. Ferreira, G.R.S.; Brito, J.S.; Procópio, T.F.; Santos, N.D.L.; de Lima, B.J.R.C.; Coelho, L.C.B.B.; Navarro, D.M.A.F.; Paiva, P.M.G.; Soares, T.; de Moura, M.C.; et al. Antimicrobial Potential of *Alpinia purpurata* Lectin (ApuL): Growth Inhibitory Action, Synergistic Effects in Combination with Antibiotics, and Antibiofilm Activity. *Microb. Pathog.* **2018**, *124*, 152–162, https://doi.org/10.1016/j.micpath.2018.08.027.
39. Lai, C.-S.; Ponnusamy, Y.; Lim, G.-L.; Ramanathan, S. Antibacterial, Antibiofilm and Antibiotic-Potentiating Effects of a Polyphenol-Rich Fraction of *Dicranopteris linearis* (Burm.f.) Underw. *J. Herb. Med.* **2021**, *25*, 100419, https://doi.org/10.1016/j.hermed.2020.100419.
40. Dias-Souza, M.V.; dos Santos, R.M.; Cerávolo, I.P.; Cosenza, G.; Ferreira Marçal, P.H.; Figueiredo, F.J.B. *Euterpe oleracea* Pulp Extract: Chemical Analyses, Antibiofilm Activity against *Staphylococcus aureus*, Cytotoxicity and Interference on the Activity of Antimicrobial Drugs. *Microb. Pathog.* **2018**, *114*, 29–35, https://doi.org/10.1016/j.micpath.2017.11.006.
41. Neto, J.; Cabral, V.; Nogueira, L.F.; da Silva, C.; Sá, L.; da Silva, A.; da Silva, W.M.; Silva, J.; Marinho, E.S.; Cavalcanti, B.C.; et al. Anti-MRSA Activity of Curcumin in Planktonic Cells and Biofilms and Determination of Possible Action Mechanisms. *Microb. Pathog.* **2021**, *155*, 104892, https://doi.org/10.1016/j.micpath.2021.104892.
42. Deepika, M.; Thangam, R.; Sakthidhasan, P.; Arun, S.; Sivasubramanian, S.; Thirumurugan, R. Combined Effect of a Natural Flavonoid Rutin from *Citrus sinensis* and Conventional Antibiotic Gentamicin on *Pseudomonas aeruginosa* Biofilm Formation. *Food Control* **2018**, *90*, 282–294, https://doi.org/10.1016/j.foodcont.2018.02.044.
